# Applications of Multifunctional Hydrogel in Tissue Engineering and Regenerative Medicine

**DOI:** 10.1002/mco2.70602

**Published:** 2026-01-29

**Authors:** Jieran Lyu, Xuemiao Liu, Qiqi Yang, Yuchang Zhang, Xing Wang

**Affiliations:** ^1^ Beijing National Laboratory for Molecular Sciences Institute of Chemistry, Chinese Academy of Sciences Beijing China; ^2^ Department of Cardiovascular Medicine Osaka University Graduate School of Medicine Suita Japan; ^3^ Department of Bone & Joint First Affiliated Hospital of Dalian Medical University Dalian China; ^4^ Department of Sports Medicine Beijing JiShuiTan Hospital Capital Medical University Beijing China; ^5^ University of Chinese Academy of Sciences Beijing China

**Keywords:** dressing, hydrogel, regenerative medicine, tissue engineering, wound healing

## Abstract

Hydrogels, with excellent hydrophilicity and high‐water content, have emerged as highly versatile biomaterials for tissue engineering and regenerative medicine. On account of the natural mimicry of extracellular matrix (ECM), moisture retention, porosity, biocompatibility, biodegradability, and tunable functionality, they provide crucial structural and biochemical support for tissue repair. As chronic wounds, aging, and degenerative diseases continue to increase, hydrogels offer great potential to overcome the limitations of traditional therapies. Despite these developments, there remains a crucial need for hydrogels that can effectively address the complex, multiphase nature of tissue repair while being cost‐effective and easily applicable in various clinical settings. This review begins by taking wound healing as a representative example, particularly elaborating on the process of wound healing and therapeutic strategies to illustrate the importance of hydrogel design by tissue engineering technology. We then comprehensively evaluate the emerging hydrogel systems that integrate multiple therapeutic functions, including drug delivery, infection prevention, stimulus responsiveness, and clinical translation for wound dressings. Additionally, this review further extends to the application scope and incorporates the latest research advancements of multifunctional hydrogels in other biomedical applications. Finally, we summarize the shortcomings of existing studies and propose future research directions, with a view to providing a valuable reference basis for the development of multifunctional hydrogels within the realm of tissue engineering and regenerative medicine.

## Graphical abstract

Schematic illustration showing multifunctional hydrogels in the application of tissue engineering and regenerative medicine.

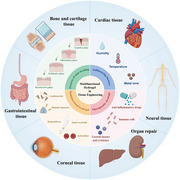

## 1 Introduction

The lack of potential donors for organ transplantation has instigated researchers across the globe to find alternatives to combat the ever‐increasing demand for organs. The past two decades have addressed this widespread issue by developing and integrating highly biocompatible yet sensitive materials for tissue regeneration. Hydrogels, with three‐dimensional hydrophilic polymer networks and high‐water content (>90%), have emerged as versatile biomaterials for tissue regeneration (e.g., wound healing, bone and cartilage repair) due to unique performances on natural mimicry of extracellular matrix (ECM), moisture retention, porosity, biocompatibility, biodegradability, and tunable functionality [1, 2]. Historically, hydrogel applications in wound care originated from the recognition of a moist environment. Dr. George Winter's pioneering research in 1962 demonstrated that occlusive dressings accelerated the epithelialization process by 50% compared with air exposure, revolutionizing the clinical practice [3]. This discovery spurred the development of hydrocolloids and hydrogels, which maintain wound hydration, facilitate cell migration, and protect against secondary infection [4, 5].

Over the past decade, significant advances in regenerative medicine have focused on the pursuit of sophisticated hydrogel biomaterials to promote tissue regeneration and prevent complications. Contemporary hydrogels have evolved beyond passive moisture retention to integrate multifunctional capabilities, including stimuli responsiveness (e.g., temperature, pH, light, enzyme, magnetic, and electric fields), controlled drug delivery, and immunomodulation [6]. Despite significant advancements, critical limitations have hindered hydrogel translation to clinical practice. For example, biodegradation kinetics remain poorly matched to tissue repair timelines: natural polymers (e.g., hyaluronic acid, collagen) degrade rapidly via enzymatic cleavage (days to weeks), whereas synthetic polymers (e.g., polyethylene glycol, polyvinyl alcohol) often persist excessively, triggering the foreign body reactions [7]. Mechanical properties are inadequate for load‐bearing applications; conventional hydrogels lack the tensile strength required for bone and cartilage regeneration, necessitating reinforcement with ceramics or nanomaterials [8]. Biological complexity by oxidative stress, dysregulated macrophage polarization, and antibiotic‐resistant demands hydrogels with integrated antibacterial, antioxidant, and pro‐angiogenic functionalities [9]. Additionally, scalability issues and regulatory hurdles for advanced formulations have restricted commercialization, with only ∼10% of preclinical studies advancing to human trials [10]. Consequently, the development of multifunctional hydrogels with elaborate design and targeted repair capabilities holds extremely high research value and clinical significance for tissue engineering and regenerative medicine.

**FIGURE 1:**
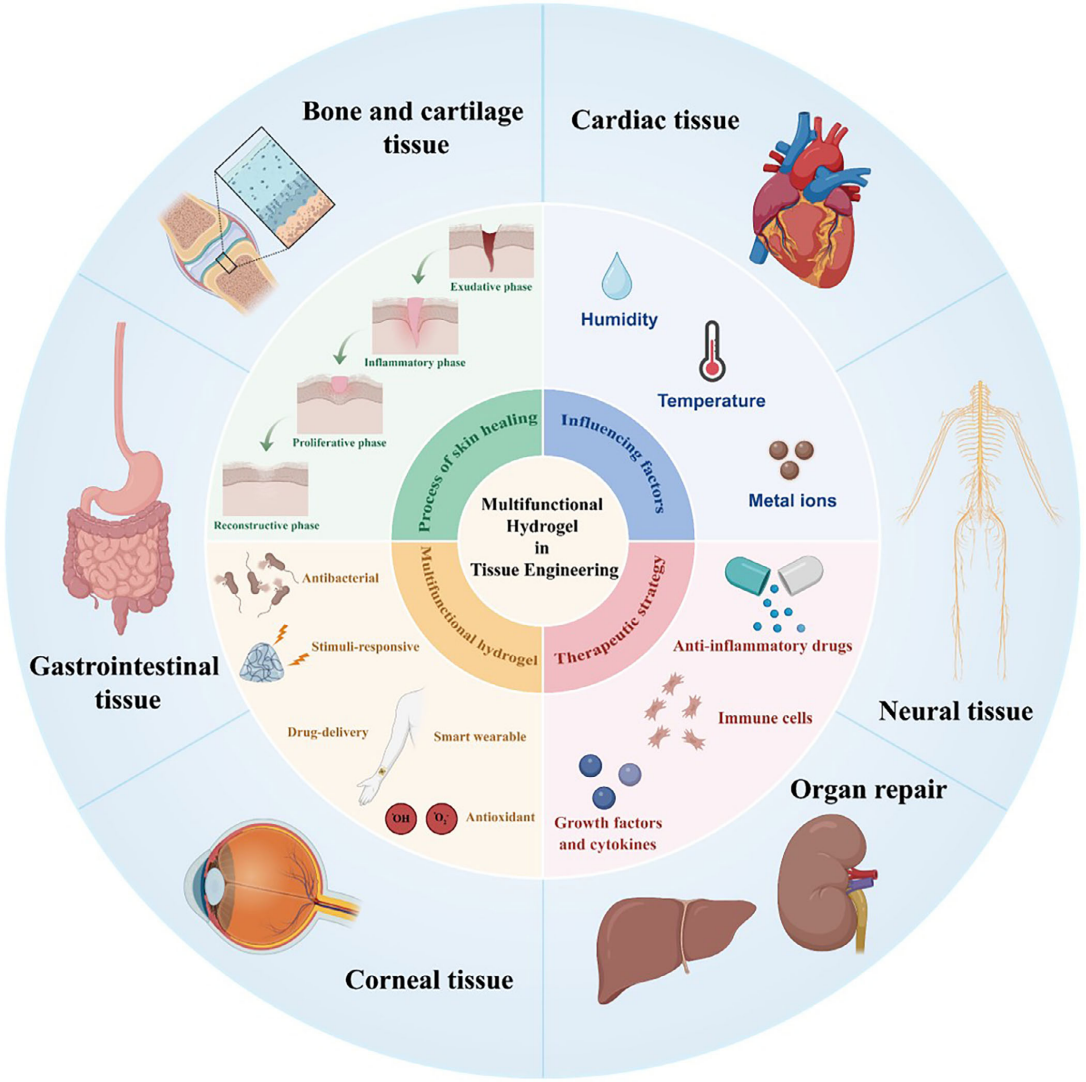
Schematic overview of multifunctional hydrogels in applications of tissue engineering and regenerative medicine.

This review synthesizes cutting‐edge research on multifunctional hydrogels to address these challenges, with a focus on translational relevance across tissue engineering applications (Figure 1). This narrative begins by presenting a representative example of the systematic analysis of the multifunctional hydrogels in the application of skin wound healing. It delves deeply into the consideration of the dynamic interplay of the healing process, ECM remodeling, and regulatory factors such as humidity, temperature, and metal ions that influence reparative outcomes. Building on this foundation, it analyzes multifunctional hydrogel‐based therapeutic strategies, distinguishing between passive approaches and active interventions to optimize the wound immune microenvironment. Then, the core focus elaborates in detail the functional design of the hydrogel specifically designed for wound healing demands, including antibacterial, hemostatic, self‐healing, and stimulus‐responsive systems. Thus, it proposes a comprehensive perspective and universal strategy that encompasses hydrogel structure and function design, influence factors, biological mechanisms, and multifaceted functions of wound healing, which provides a reference basis for in‐depth thinking and meticulous design in other fields of tissue engineering and regenerative medicine. Under these circumstances, this review further extends to the application of multifunctional hydrogel in bone and cartilage regeneration, cardiac repair, neural tissue regeneration, corneal tissue repair, visceral organ repair, and gastrointestinal tissue regeneration, thereby highlighting the cross‐tissue adaptability through structural and biochemical customization. Finally, it concludes by critically examining unresolved challenges (degradation tuning, mechanical reinforcement, clinical scalability) and outlining future directions for advancing hydrogel‐based therapies from bench to bedside. By integrating materials science, immunology, and clinical perspectives, this review also aims to underscore the transformative potential of multifunctional hydrogels in reshaping regenerative medicine.

## 2 Skin Wound Healing

**FIGURE 2:**
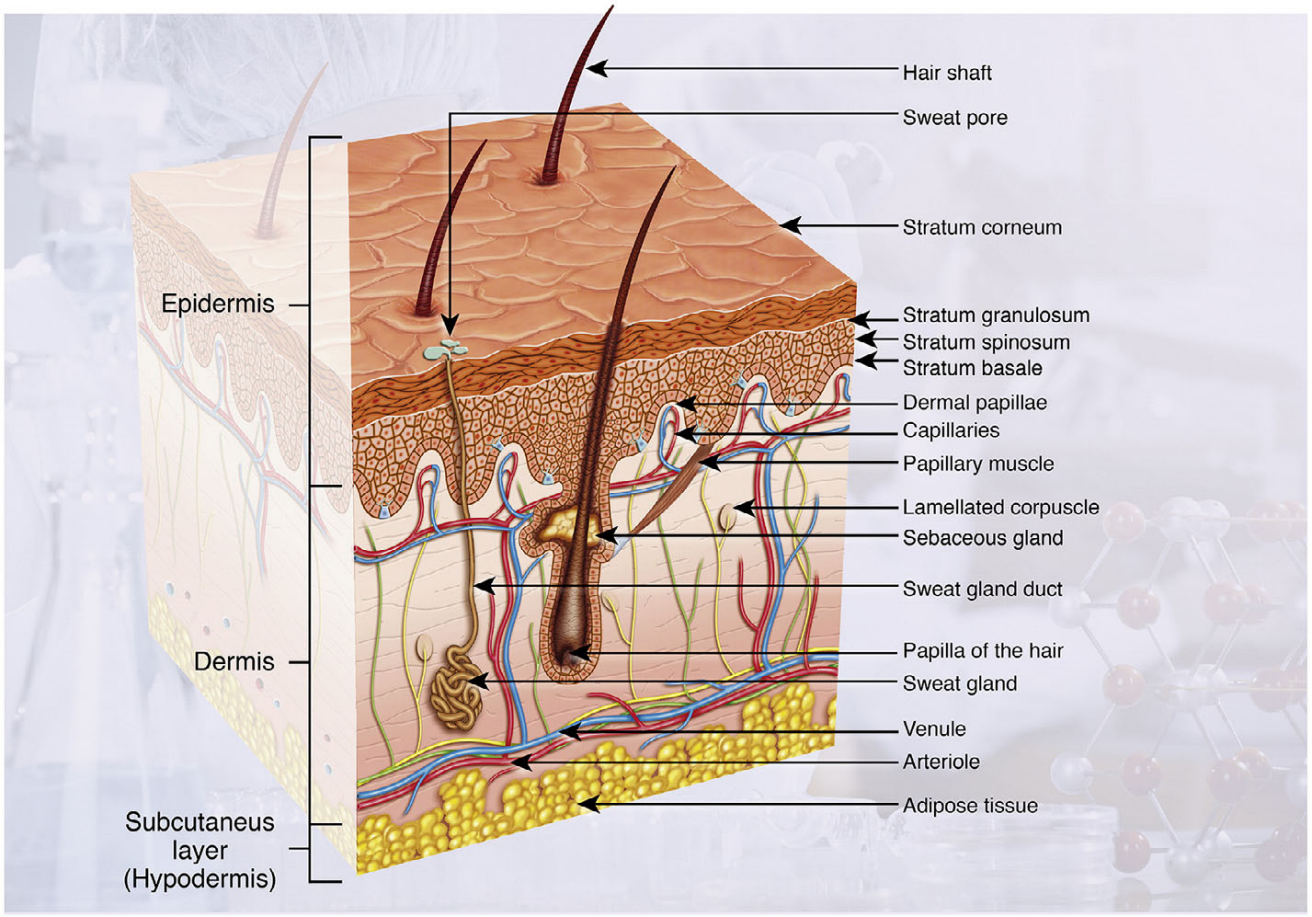
Schematic picture of the native skin that is subclassified into three main compartments: epidermis, dermis, and subcutis (hypodermis). *Source*: Reproduced with permission from [16]. Copyright 2014, Elsevier.

The skin, the body's largest organ, comprises a sophisticated hierarchical structure consisting of the epidermis, dermis, and subcutaneous tissue, which collectively perform multifaceted functions including physical barrier protection, water homeostasis maintenance, sensory perception, and immunological defense [11, 12]. The epidermis, the outermost layer, primarily consists of keratinocytes (80% of cellular components) alongside specialized cell populations such as melanocytes, Langerhans cells, and Merkel cells, which, respectively, regulate the photoprotection, immune surveillance, and neurosensory transduction [13]. Beneath the epidermis lies the dermis, a connective tissue network composed of fibroblasts, immune cells, and ECM components (collagen and elastic fibers), which provides structural integrity and houses vasculature, nerve endings, and appendages like hair follicles and sweat glands [14]. The subcutaneous tissue, rich in adipocytes and vascular networks, serves as an energy reservoir and thermoregulatory buffer for the body surface. The multilayered structure of the skin is shown in Figure 2, and this complex architecture is frequently disrupted by external insults (mechanical trauma, thermal/chemical injury) or internal pathologies (diabetic ulcers, vascular dysfunction), leading to the acute or chronic wounds that compromise physiological homeostasis [15, 16].

The clinical management of wounds remains a pressing global challenge, particularly with the rising prevalence of chronic nonhealing conditions. Acute wounds typically resolve within 10–20 days through an orderly reparative process involving hemostasis, inflammation, proliferation, and remodeling [17, 18, 19]. In contrast, chronic wounds generally associated with diabetes, venous insufficiency, or pressure ischemia exhibit prolonged inflammation (>3 months), dysregulated angiogenesis, and bacterial biofilm formation, resulting in high morbidity, disability rates, and mortality [20]. Diabetic foot ulcers (DFUs), a paradigmatic chronic wound, affect approximately 6.3% of the global diabetic population, with an amputation occurring every 30 s and a 5‐year mortality rate of 40%–70% [21, 22]. The cost of treatment has become a global issue, affecting nearly 5.7 million people at a cost of $20 billion per year in the USA alone; a report in the United Kingdom states that treatment and care of chronic wounds accounts for at least 5.5% of total NHS spending [23, 24]. Even if some skin wounds can heal, they can bring about hyperplastic scars or keloids, which not only lead to functional deficiencies and affect aesthetics, but also adversely affect the patient's psychology [25]. In this context, scars and associated functional and aesthetic problems are also a huge healthcare burden. The global scar treatment market is expected to reach approximately $32 billion by 2027. As a result, skin wounds cause immense physical and psychological trauma to patients, severely affecting normal social life and imposing a huge financial burden on individuals and society, further underscoring the need for the development of advanced regenerative strategies.

### 2.1 Process of Skin Wound Healing

**FIGURE 3:**
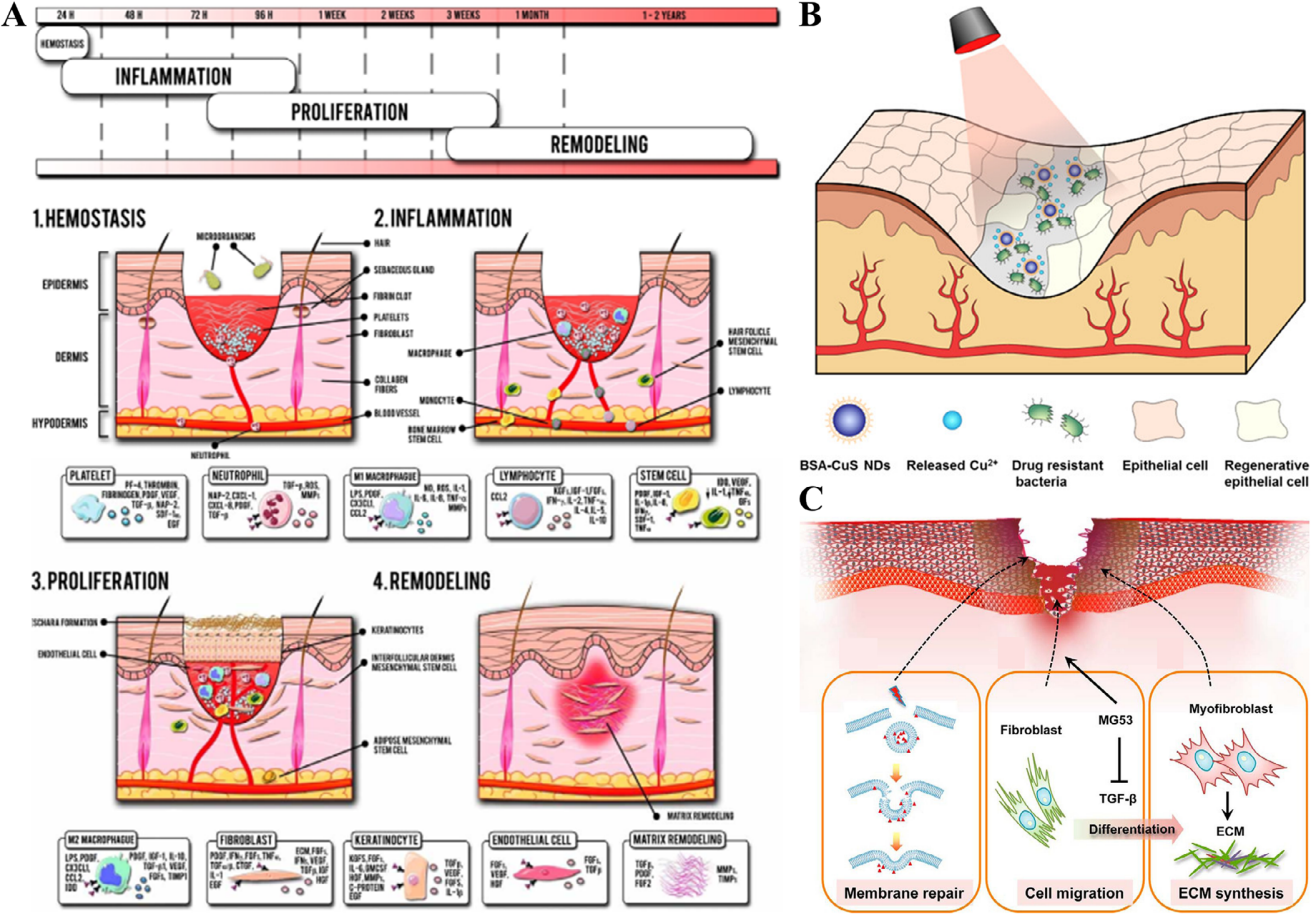
Process of skin wound healing. (A) Normal wound healing steps: hemostasis, inflammation, proliferation, and remodeling. In cell boxes: the left‐handed cytokines, GFs, etc., stimulate cells, and the right‐handed ones are secreted by these cells. *Source*: Reproduced with permission from [28]. Copyright 2020, Elsevier. (B) Schematic diagram and effect of CuS NDs with a dual functional nanosystem to cure multidrug‐resistant bacteria‐infected chronic nonhealing wounds. *Source*: Reproduced with permission from [45]. Copyright 2019, ACS Publications. (C) Schematic illustrating the role of MG53 in wound healing and scar formation. Left: MG53 nucleates the cell membrane repair machinery and protects against acute injury to keratinocytes and fibroblasts in response to dermal injury. Middle: MG53 promotes the migration of fibroblasts to the wound sites by modulating stress fiber formation. Right: MG53 regulates TGF‐β/Smad signaling to control the differentiation of fibroblasts into myofibroblasts, leading to down‐regulation of ECM proteins during the tissue remodeling process of wound healing. Reproduced with permission from [46]. Copyright 2015, American Society for Biochemistry and Molecular Biology.

As the most external organ of the body, the skin is vulnerable to microbial, accidental, or surgical injury. Large wounds or chronic wounds (e.g., diabetic ulcers) have a long healing cycle and are difficult to heal on their own, often requiring medical intervention such as surgical suturing, artificial skin grafts, or functional dressings [26]. The treatment of infected wounds, in particular, should focus on the role of bacteria or biofilms, which is currently the main difficulty and research hotspot in the field of medicine [27]. Microscopically, it appears that skin wound healing is in fact a dynamic process of a multicellular population, mainly involving interactions between a variety of cells and the ECM. The healing process experienced after a wound occurs consists of four main phases, namely, exudative, inflammatory, proliferative, and reconstructive phases, with no clear boundaries between each of these phases [28, 29]. The various stages of skin wound healing and the corresponding physiological changes are shown schematically in Figure 3A.

The exudative phase is the initial stage of wound repair, which is mainly characterized by extravasation of blood and tissue fluid. The densely distributed capillary network in human skin will trigger physiological bleeding when damaged, and this exudative response actually has multiple protective mechanisms: first, the outflow of blood can effectively remove foreign bodies and pathogenic microorganisms on the wound surface through physical flushing; second, platelets in the blood components are rapidly activated upon contact with the wound surface, releasing coagulation factors and initiating a cascade reaction, which prompts the conversion of fibrinogen to fibrin network, forming a solid blood clot. This dynamic process not only controls continuous blood loss but also creates a favorable microenvironment for subsequent tissue repair. The inflammatory phase usually occurs in tandem with hemorrhage and involves primarily cellular and vascular responses. During this process, histamine and 5‐hydroxytryptamine released from the exudate cause vasodilatation, which provides conditions for macrophages to remove necrotic cells from the tissue. This series of changes not only leads to a change in pH at the wound site but also causes swelling and a constant feeling of pain [30, 31]. During the proliferative phase, fibroblasts and other basal cells proliferate and migrate to the skin defect, synthesize collagen, give the new tissue a certain shape and strength, and gradually form capillaries and enter granulation tissue, accelerating nutrient delivery until the wound closes [32, 33]. Finally, the remodeling phase primarily involves collagen rearrangement and connective tissue formation. During this process, the new skin tissue matures until the wound is completely healed.

The wound healing process is accompanied by a synchronized immune response, and disruption of any part of the process may lead to multiple pathologic wounds. As a result, the repair process usually presents two distinct outcomes: a regenerative phase, in which damaged cells are replaced by the same type of cells, leaving no lasting traces of damage; and fibroproliferation or fibrosis, in which connective tissue replaces normal parenchymal tissues, leading to extensive remodeling of the ECM and the formation of permanent, nonfunctional scar tissue. In addition, a dysregulated inflammatory response perpetuates the inflammatory cycle, which in turn leads to tissue damage, ulceration, and continued growth of pathogens, creating chronic wounds that are difficult to heal. Most chronic wounds are often accompanied by infection and microbial film formation. If not treated in time, chronic wounds can lead to amputation, organ failure, and even death. Therefore, inflammation plays a crucial role in wound healing, not only in the body's response to the danger signals generated after injury, but also in the maintenance of homeostasis within the body. Improving the inflammatory microenvironment and promoting wound healing has become a central factor in the design of wound repair materials.

### 2.2 Influencing Factors of Skin Wound Healing

Skin wounds are essentially interruptions of a continuous cell layer, and the healing process is a dynamic one in which cells eventually fill the gap by proliferating and migrating toward the missing area. Wound healing is not only regulated by various protein factors and cells within the organism, but also influenced by the external microenvironment, such as microorganisms, pH, metal ions, temperature, air pressure, and humidity. This review will focus on the role of abiotic factors such as humidity, temperature, and metal ions in the wound healing process.

#### 2.2.1 Humidity

Early on, it was widely recognized that keeping the skin environment dry facilitated wound healing. However, it wasn't until 1962 that Dr. Winter proposed a wet healing mechanism for skin wounds. His research found that keeping wounds moist promoted faster healing and was less likely to form scars than exposing wounds to air. This discovery turned conventional wisdom on its head and provided a new way of thinking about wound care and treatment [3]. The specific reason was that without the skin, the cells that were originally beneath the skin were exposed to the outermost layer, in direct contact with the air. In dry environments, cells rapidly lose water, dry out, and crust, which allows the surrounding newborn cells to migrate only from underneath the crust during the healing process, greatly limiting the rate of migration. For wounds that remain moist, cells can migrate directly through the moist surface, which greatly reduces the time required for wound healing. So, the selection of wet dressings for wound care has significant advantages, prompting the research and development of wet dressings such as hydrocolloids and hydrogels to receive widespread attention and become an important research direction [34, 35, 36].

#### 2.2.2 Temperature

Temperature has long been recognized as one of the most important factors affecting skin wound healing [37]. For all types of acute or chronic wounds, the initial period of healing is usually characterized by a slight increase in wound temperature due to the inflammatory response, followed by a transition to a plateau below normal body temperature (32°C) [38]. Studies have shown that the activity of cells such as skin‐associated keratinocytes is significantly reduced when the temperature is below 33°C, and therefore 33°C is considered the critical temperature for cellular bioactivity [39]. Prolonged exposure of the wound temperature below this threshold leads to a decrease in the number of fibroblasts and late inflammatory cells, and impaired collagen deposition, thus prolonging the healing time of the wound [40]. To overcome the adverse effects of low temperature on wound healing, appropriate heat treatment can effectively promote wound healing. Appropriately elevated temperatures help to increase the transport of oxygen by the blood in the capillaries, as well as the activity of the stromal cells. A prospective single‐center randomized clinical trial evaluated the efficacy of a 38°C thermostatic radiant heat dressing in patients with Stage 3 and 4 pressure ulcers. Enrolling patients with chronic wounds, the intervention accelerated wound healing despite the heat therapy group being generally frailer than the standard treatment group. Specifically, compared with standard care, the heat therapy group achieved reductions to 75% (6.4 days earlier, *p* = 0.057), 50% (9.6 days earlier, *p* = 0.039), and 25% (7.2 days earlier, *p* = 0.01) of the original wound area [41]. Therefore, increasing the wound temperature appropriately and designing and developing new thermostatic dressings can effectively accelerate the wound healing process.

#### 2.2.3 Metal Ions

The human body cannot grow and develop without metal elements such as iron, copper, zinc, manganese, and nickel, which play a vital role in bone metabolism, the central nervous system, immune function, and wound healing. Among them, copper and zinc have been shown to play a more active role in skin wound healing [42]. Several studies have demonstrated that copper ions are potent stimulators of endothelial activity, promoting the proliferation of umbilical artery and vein endothelial cells, which play an important role in angiogenesis [43]. In addition, nonangiogenesis‐stimulating substances such as heparin and tripeptide, when combined with copper ions, are also capable of having pro‐angiogenic effects [44]. During skin wound healing, the vascularization of new tissue provides more adequate nutrients to the wound, thus accelerating wound healing. However, the biological effect of copper ions is strongly concentration‐dependent. At physiological levels (typically <50 µM), copper promotes endothelial proliferation and angiogenesis, while excessive concentrations (>100 µM) may lead to oxidative stress, mitochondrial dysfunction, and cytotoxicity to the endothelial and epithelial cells. Therefore, defining the therapeutic window of copper ion exposure is crucial for safe clinical application. Not only that, but copper ions also show significant advantages in inhibiting bacterial activity. For example, Qiao et al. [45] developed ultrasmall copper sulfide nanodots (CuS NDs) for methicillin‐resistant *Staphylococcus aureus* (MRSA)‐infected diabetic mice wound treatment (Figure 3B), and the results showed that the photothermal effect of the material effectively suppressed the drug‐resistant bacteria under near‐infrared light irradiation, and the released copper ions significantly promoted the wound's vascularization ability. After 12 days of treatment, the wound area in the experimental group of mice was reduced to about 3%, while in the control group it remained at about 90% of the initial area. Similarly, zinc plays an important role in the skin repair process, and its effects are also dose‐dependent. Physiological concentrations of zinc (5–50 µM) have been reported to enhance platelet activity and aggregation for rapid hemostasis, inhibit the expression of several inflammatory factors, and promote keratinocyte migration and re‐epithelialization. Nevertheless, excessive zinc (>100 µM) may impair fibroblast proliferation and delay wound healing. Additionally, MG53 is a trimeric protein involved in cell membrane repair, and zinc ions act as its key cofactor (Figure 3C) [46, 47]. By enhancing cell membrane repair ability mediated by MG53, promoting fibroblast migration to the wound site, and regulating the TGF‐β signaling pathway to inhibit myofibroblast differentiation, zinc ions could work in concert with MG53 to accelerate wound healing and reduce scar formation. In addition to copper and zinc, other metallic elements such as magnesium, aluminum, iron, and strontium also have a role in the skin repair process [48, 49]. Taken together, the appropriate addition of suitable metal ions at safe and effective concentrations can significantly promote the repair and remodeling of skin wounds.

### 2.3 Hydrogel‐Based Therapeutic Strategies for Skin Wound Healing

**FIGURE 4:**
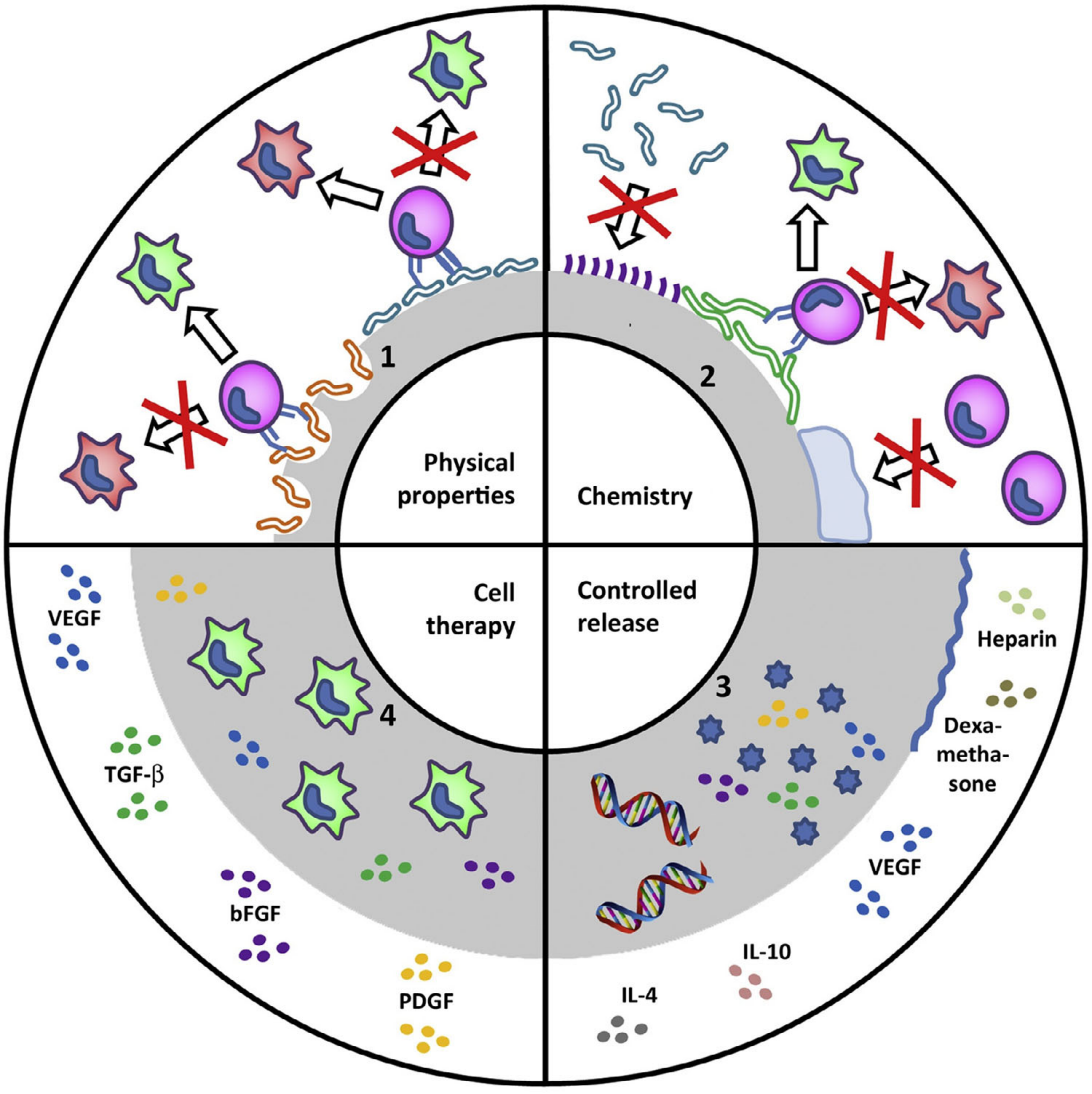
Schematic representation of different strategies that can be used to achieve immunomodulation around implants and tissue‐engineered structures. The activation of monocytes into mature tissue macrophages, among other host inflammatory cells, will play a central role in tissue remodeling and integration at the biomaterial‐host interface. *Source*: Reproduced with permission from [57]. Copyright 2016, Elsevier.

In the last decades, several methods of treating chronic wounds have been investigated for the different stages of wound healing, including different types of dressings, delivery of cytokines and growth factors, cellular therapy, and electrical or mechanical stimulation therapies [50, 51, 52, 53, 54, 55, 56]. Among these techniques, hydrogel biomaterial is considered one of the most effective strategies, acting as a temporary substance capable of supporting the whole process of wound healing. Therefore, several hydrogel‐based wound dressings have been developed in recent years to mimic the skin microenvironment. Strategies to modulate the immune microenvironment around the skin mainly include cellular therapeutic approaches by altering the physical and chemical properties of the hydrogel host interface, as well as controlling the release of anti‐inflammatory or proinflammatory cytokines, or even directly encapsulating immune cells or inducing cell entry, which work together to optimize the immune response and promote wound repair and regeneration (Figure 4) [57].

#### 2.3.1 Passive Therapeutic Strategies

The field of hydrogel has long focused on the study of biologically inert materials, and passive therapeutic strategies for such materials typically involve avoiding cell‐material interactions by altering their intrinsic physicochemical properties, thereby minimizing the host immune response. Currently, strategies for designing such hydrogel biomaterials include directly altering the physical or chemical properties by incorporating molecules or matrices capable of targeting specific cells. These strategies aim to make hydrogels more adaptable to the host environment while reducing possible immune rejection [58].

**FIGURE 5:**
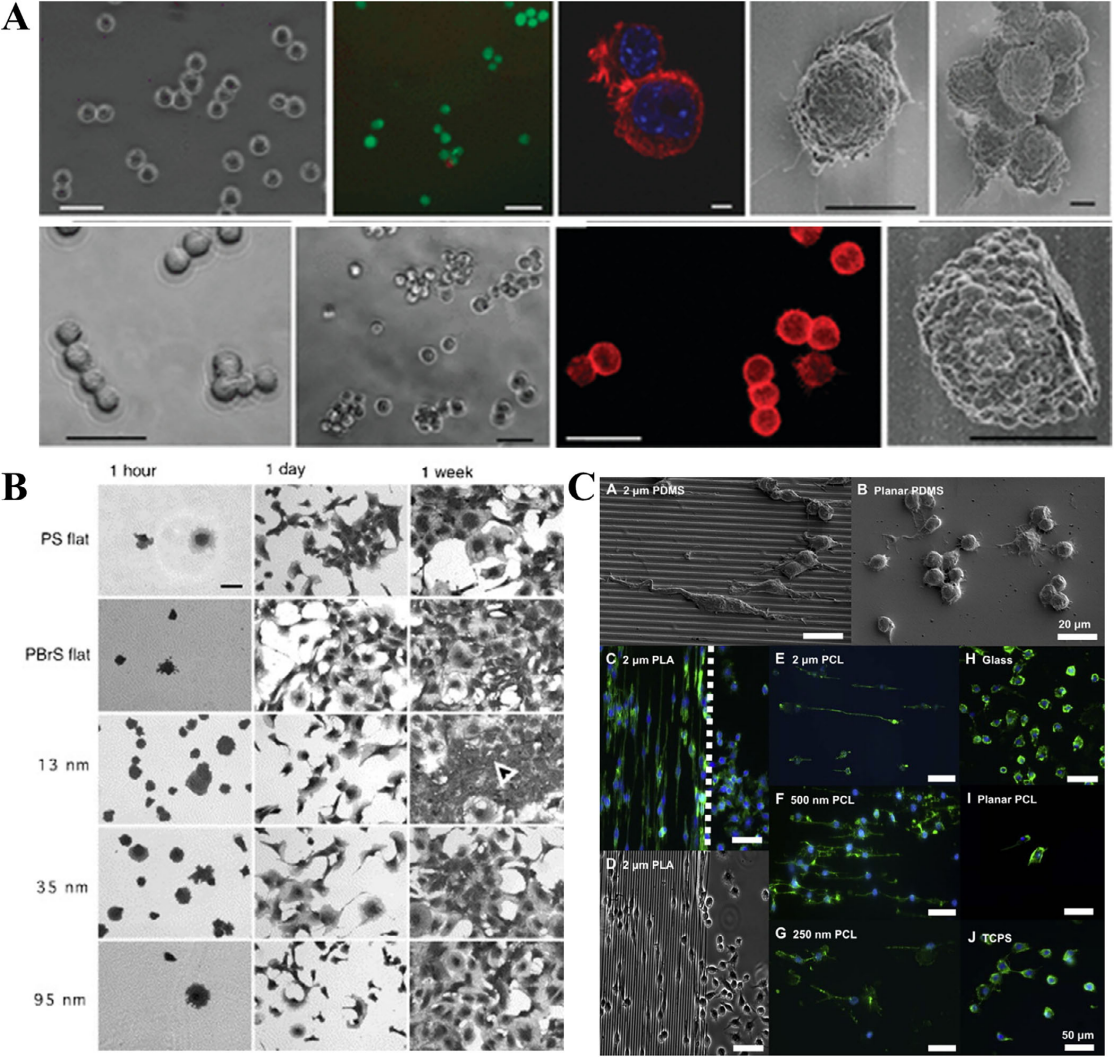
Biomimetic morphology strategies. (A) L929 Fibroblasts cultured on unpatterned starPEG substrates after 24 h (upper row) and 48 h (lower row). Some cells adhered to the substrate and stayed in a round shape, while a tendency of cluster formation was observed. *Source*: Reproduced with permission from [62]. Copyright 2009, ACS Publications. (B) Coomassie blue histology of human endothelial cell line (HGTFN) cultured on the controls and test surfaces at 1 h, 1 day, and 1 week time points. It is noted that at the 1‐week time point, cells on the 13 nm islands formed multilayers (arrowhead). PS: polystyrene; PBrS: poly(4‐bromostyrene). *Source*: Reproduced with permission from [63]. Copyright 2003, Elsevier. (C) Macrophage morphology changes on topographical gratings compared with planar controls. RAW 264.7 cultured on 2 µm PDMS gratings for 48 h showed that cells elongated in the direction of the gratings, but maintained their native round morphology on the planar PDMS control. PDMS: poly(dimethyl siloxane); PLA: polylactic acid; TCPS: tissue culture polystyrene. *Source*: Reproduced with permission from [64]. Copyright 2010, Elsevier.

An effective strategy for directing cellular responses to hydrogels is alteration of the physical properties, such as surface roughness and morphology, which play a crucial role in protein adsorption and immune cell responses. Nanoscale morphological changes can alter the activity of adsorbed proteins via conformational changes upon the adsorption [59]. In natural environments, cellular responses to ECM components, especially at the nanoscale, are characterized by cell adhesion, proliferation, migration, and gene expression. Therefore, mimicking or directly using the ECM structure can create a favorable microenvironment for wound healing. For example, Kajahn et al. [60] demonstrated that the presence of high‐sulfated hyaluronic acid (HA) significantly interfered with interleukin‐6 (IL‐6), interferon gamma (IFN‐γ), and monocyte chemotactic protein (MCP‐1)‐mediated M1 macrophage activation, and even induced the secretion of M2 macrophage‐associated cytokine, interleukin‐10 (IL‐10). This artificial ECM could be used as a coating to help reduce inflammation, prevent M1 macrophage activation around the implants, and promote wound healing. In addition, micrometer‐ and nanometer‐scale patterns of simulated ECM morphology imprinted on material surfaces can affect the function of fibroblasts, epithelial cells, and endothelial cells (Figure 5) [61, 62, 63, 64]. These studies suggest that by modulating the physical properties of hydrogels, immune responses can be effectively modulated to promote wound healing and tissue repair [65].

**FIGURE 6:**
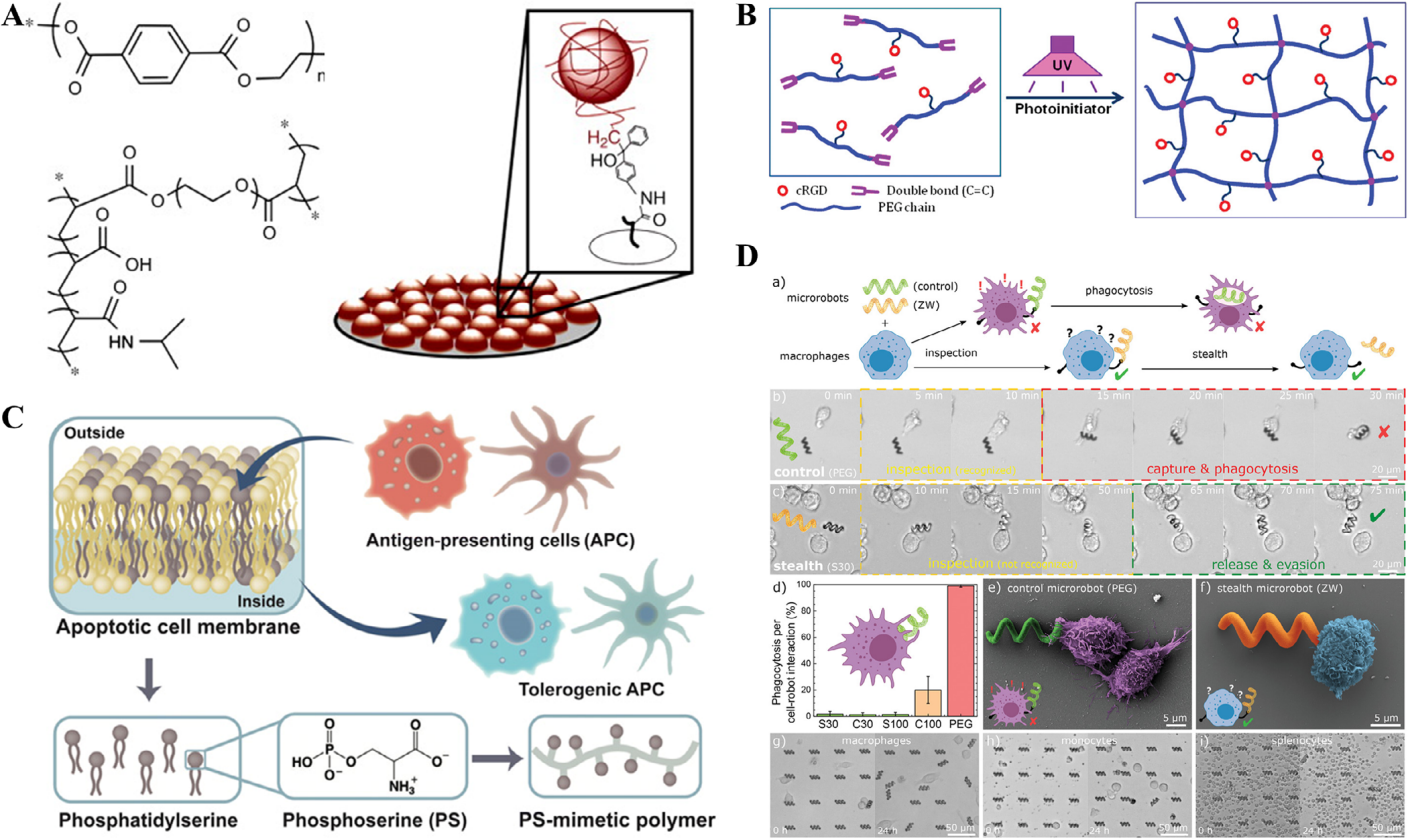
Passive nonbiofouling strategies. (A) A coating strategy based on thin films of poly(*N*‐isopropylacrylamide) (pNIPAM) hydrogel microparticles cross‐linked with poly (ethylene glycol) diacrylate. *Source*: Reproduced from [69] with permission. Copyright 2008, Elsevier. (B) A type of bioactive poly (ethylene glycol) diacrylate macromers, cyclic Arg‐Gly‐Asp (cRGD)‐PEGDA, mimicking the cell‐adhesive properties of ECM. *Source*: Reproduced with permission from [73]. Copyright 2009, ACS Publications. (C) Design of phosphoserine (PS)‐mimetic polymers built from phosphoserine, an immunomodulatory molecule naturally occurring on the outside membranes of apoptotic cells. *Source*: Reproduced with permission from [76]. Copyright 2020, AAAS. (D) Nonimmunogenic stealth microrobots. (a) Schematic of cell–robot interaction: control microrobots are captured and phagocytosed by activated macrophages, while undetected stealth microrobots are released after inspection. (b) Inspection and capture of control PEG microrobots. After a short inspection time, the microrobot is recognized by the macrophage, captured, and phagocytized. (c) Inspection and release of stealth microrobots. After exhaustive inspection, the stealth microrobot is not recognized, and it is released by the macrophage, avoiding phagocytosis. (d) Phagocytosis rate for different types of microrobots (normalized by cell–robot interactions). The error bars represent the standard deviation. (e, f) SEM image of macrophage interacting with a nonstealth control robot (e) and with a zwitterionic stealth microrobot (f) at the early stages of inspection. (g) Macrophages, (h) monocytes, and (i) splenocytes interacting with arrays of zwitterionic stealth microrobots for 24 h. *Source*: Reproduced with permission from [77]. Copyright 2020, Wiley.

Additionally, modifying the surface chemistry of hydrogels is a direct way to modulate protein adsorption and cell behavior, and limiting adhesion to cells, activation, and restriction of macrophage fusion into foreign‐body giant cells by modulating the surface chemistry of hydrogels is another strategy for directing cellular responses. The type, level, and conformation of serum proteins adsorbed to the surface of hydrogels depend on the terminal chemistry [66]. Typically, adsorbed protein layers provide binding sites for protein‐specific receptors (integrins) on polymorphonuclear granulocytes, monocytes, and macrophages, and thus, modulation of the surface chemical properties of the protein layer may allow it to bind to different receptors in the immune cells and to participate in signaling, thus altering cellular responses. Indeed, comprehensive proteomics studies have shown that the macrophages change their protein expression and cytokine/chemokine response when cultured on surface‐modified hydrophobic, hydrophilic, and/or ionic polymers [67]. A study suggested that macrophages attached to the hydrogel surface provided fewer integrin‐binding sites, which reduced the integrin‐mediated cell spreading and led to macrophage apoptosis [68]. These findings provided guidance for surface modifications of hydrogels that induce the desired polymorphonuclear granulocyte and macrophage activity. Thus, the need to mitigate host inflammatory responses to implanted hydrogels has led to the development of “immuno‐isolating” scaffolds, such as dense hydrophilic polymer membranes and brushes without bio‐contamination to reduce foreign body reactions by reducing protein adsorption, leukocyte activation, and adhesion. Semipermeable hydrogels are widely used in the study of bio‐contamination‐free implantable coatings due to their high‐water content, ease of transportation, and the possibility of further chemical modification with different reactive groups (Figure 6A) [69, 70, 71]. Bridges et al. [72] reported a coating strategy using poly (N‐isopropylacrylamide) (pNIPAM) films made of microgel particles with polyethylene glycol (PEG) short‐chain cross‐linking. The hydrogel effectively fills the inhomogeneity of the surface of the model substrate, prevents primary human macrophage adhesion in vitro, and attenuates acute‐phase leukocyte adhesion and proinflammatory cytokine levels in vivo. In addition, binding of integrin ligands can endow the hydrogel surfaces with antibiofouling effects (Figure 6B) [73, 74]. The surfaces modified with PEG can resist protein adsorption, prevent passive cell attachment and subsequent cell activation, and prevent nonspecific cell‐material interactions, while integrin adhesion sites on the biomaterial surfaces facilitate the induction of specific cell activation. Although protein adsorption and cell adhesion can be somewhat reduced by altering the surface chemistry and physical properties of hydrogels, they always lead to the formation of fibrous envelopes. It is well known that almost all synthetic materials trigger a foreign body reaction and formation of a fibrous capsule within 1 month after implantation, and studies have shown that superhydrophilic amphiphilic hydrogels have great potential to solve this problem. Jiang et al. investigated that a zwitterionic polycarboxybetaine (pCB) hydrogel could avoid the formation of a fibrous capsule after subcutaneous implantation in mice for 3 months, which was the longest time for hydrogel scaffolds to alleviate foreign body reactions [75]. Then, they further designed a nonfouling amphiphilic phosphoserine mimetic polymer with the built‐in immunomodulatory functions, which provided new insights into the fundamental design and had far‐reaching implications for a wide range of applications such as drug delivery, implants, and cell therapy (Figure 6C) [76]. In addition, Metin Sitti et al. [77] designed a nonimmunogenic stealthy hydrogel microrobot that could avoid recognition by immune cells. It had adjustable mechanical properties, antibiological contamination ability, and nonimmunogenicity, which could be used in medical microrobotics and other bioengineering applications (Figure 6D).

Although these passive therapeutic strategies have shown better promise for in vitro use as well as during the first few days of acuity when used in vivo, they lack long‐term stability and do not show consistently favorable responses to chronic inflammation in vivo. Therefore, it is more important to control the presentation or concealment of biologically active sites of adsorbed proteins, that is, to develop more biologically active hydrogel scaffolds than to directly reduce protein adsorption, rather than to reduce the interaction of the implanted materials with the surrounding tissue.

#### 2.3.2 Active Therapeutic Strategies

Tissue repair and regeneration processes are accompanied by the removal of damaged tissue debris, revascularization, ECM remodeling, and activation and differentiation of stem cells, which are largely regulated by pro‐ and anti‐inflammatory responses and together promote functional regeneration of injured tissues. Therefore, delivery of immunomodulatory factors targeting these processes has emerged as a promising therapeutic strategy. In recent years, there have been significant advances in active therapeutic strategies based on the localized release of active substances to modulate inflammation. In particular, the use of controlled‐release drug strategies to release anti‐inflammatory and/or wound‐healing‐promoting small molecules to the wound site has been widely used to fight inflammation and promote wound repair. These strategies are achieved primarily through the release of drug targets and biomolecules, including signaling cytokines, to modulate immune responses and promote effective wound healing and tissue regeneration.

##### 2.3.2.1 Delivery of Small‐Molecule Anti‐Inflammatory Drugs

Given the wide variety of drugs used in clinical practice for the systemic modulation of inflammation, the topical administration of pharmacological agents with anti‐inflammatory properties that are released in a controlled manner from the hydrogels has been extensively studied [78]. Glucocorticoids are potent inhibitors of the immune response, they inhibit inflammatory cell activation by inhibiting the synthesis of inflammatory mediators, including several cytokines and chemokines, prostaglandins, leukotrienes, protein hydrolases, free oxygen radicals, and nitric oxide, and they also promote inflammation and adaptive immune responses by enhancing the release of anti‐inflammatory cytokines, suppressing cellular immunity (helper T cells, helper T cell, Th1‐directed) as well as enhancement of humoral immunity (Th2‐directed) and tolerance to promote the abatement of inflammation and adaptive immune responses [79]. Indeed, it has been demonstrated that coupling dexamethasone to hydrogels at the implantation site reduces implantation‐associated inflammation, as evidenced by a decrease in the number of polymorphonuclear granulocytes during the initial inflammatory phase, and a decrease in the number of macrophages, lymphocytes, and fibrous peritoneum at later stages [80]. For example, Darby et al. [81] suggested that aspirin may have a facilitating effect on the treatment of chronic wounds because of its ability to inhibit inflammatory pathways that were increased by proinflammatory cytokines, while increasing anti‐inflammatory molecules that promote repair and alleviate inflammation. However, clinical studies on the use of nonresidual anti‐inflammatory drugs for acute and chronic wound healing and pain control remain to be further carried out, and these drugs were accompanied by gastrointestinal, renal, and cardiovascular complications. Therefore, the exact effect of nonsteroidal anti‐inflammatory drugs on healing awaits further research.

##### 2.3.2.2 Delivery of Growth Factors and Cytokines

In biological inflammatory environments, complex signaling networks of biomolecules can regulate the inflammatory phenotype of macrophages. Genes for growth factors and cytokines, as bioactive components, are the most suitable candidates to promote wound healing, and they play a crucial role in all stages of wound healing. Studies have shown that secretion of key cytokines can modulate inflammatory responses and have been shown to serve as effective clinical targets. In addition, similar to anti‐inflammatory drug release approaches, many researchers have explored strategies to deliver cytokines from hydrogels to modulate macrophage behavior, with the expectation that these bioactive factors can reduce inflammatory responses and improve material‐tissue integration [82]. These strategies aim to enhance material repair by precisely modulating macrophage function to promote wound healing while reducing adverse immune responses.

**FIGURE 7:**
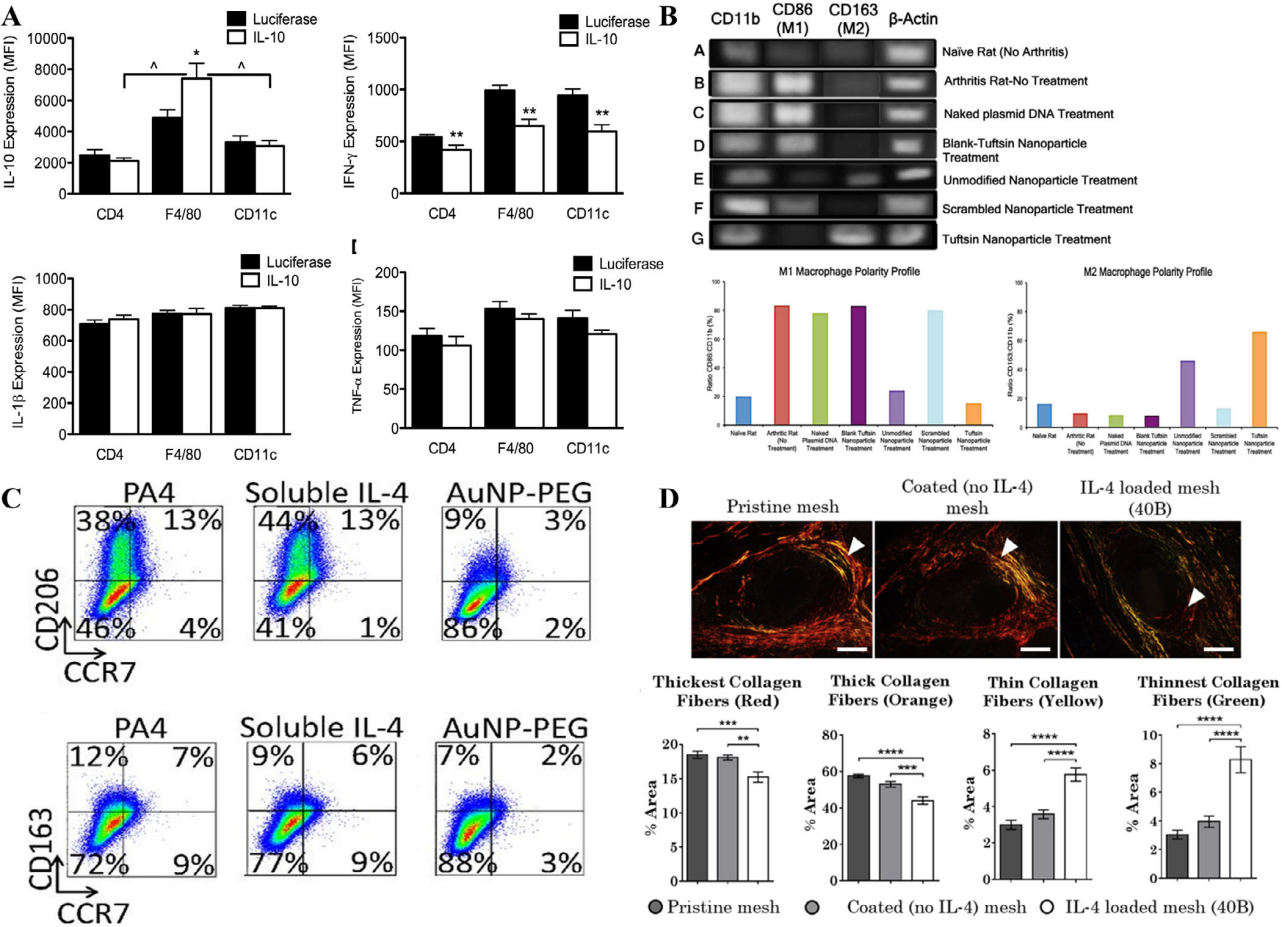
Modulating inflammatory behavior by delivering cytokines. (A) Cytokine production within IL‐10 virus‐releasing scaffolds. IL‐10, IFN‐γ, IL‐1β, and TNF‐α expression as measured by median fluorescence intensity (MFI) on CD4, F4/80, and CD11c positive cells isolated from scaffolds loaded with either luciferase or IL‐10 encoding virus. *Source*: Reproduced with permission from [88]. Copyright 2014, Elsevier. (B) Synovial macrophage polarity profile for various control and targeted nanoparticle treatments containing murine IL‐10 plasmid DNA evaluated via RT‐PCR analysis. *Source*: Reproduced with permission from [89]. Copyright 2015, Elsevier. (C) THP‐1‐derived Mφs are treated for 3 days with 20 ng/mL IL‐4 as 30‐nm PA4 or soluble IL‐4 or 30 nm AuNP‐PEG. Plots show expression of the M2a (CD206) or M2c (CD163) vs. the M1 marker (CCR7). PA4: IL‐4–conjugated particles; AuNPs: gold nanoparticles; Mφs: macrophages. *Source*: Reproduced with permission from [92]. Copyright 2018, PNAS. (D) Picro Sirius Red‐stained tissue sections of mice implanted with a piece of pristine, coated (no IL‐4) and IL‐4‐loaded (40B) mesh at 90 days. Arrowheads indicate the capsule surrounding single mesh fibers. Image analysis shows the quality of the collagen capsule and surrounding mesh fibers. *Source*: Reproduced with permission from [93]. Copyright 2017, Elsevier.

Currently, most studies focus on the delivery of known anti‐inflammatory cytokines such as interleukin‐4 (IL‐4) and IL‐10. By delivering these anti‐inflammatory factors, macrophages can be induced to polarize to an alternatively activated macrophage phenotype, which modulates the immune response and promotes wound healing. Currently, the most important and effective clinically applied strategies for cytokine therapy aimed at reducing scar formation are mainly based on the delivery of transforming growth factor‐β3 (TGF‐β3), mannose‐9‐phosphate (M6P), and IL‐10. These strategies promote a more optimal healing process by modulating macrophage function and inhibiting excessive inflammatory responses, thereby reducing scar formation [83, 84]. Also, since IL‐10 has a significant effect on various types of immune cells via promoting the regulatory T cells (Tregs) to act as anti‐inflammatory and stimulating macrophage polarization, the control of local IL‐10 concentrations has been widely used in various cytokine‐based therapeutic strategies for the treatment of chronic wounds [85]. In addition, IL‐10 has immunostimulatory effects on various cell types (e.g., T cells, B cells, and mast cells) and immunosuppressive effects on monocytes/macrophages [86]. IL‐10 also inhibits the synthesis of several cytokines (e.g., interleukin‐1 [IL‐1], interleukin‐2 [IL‐2], interleukin‐3 [IL‐3], interleukin‐6 [IL‐6], interleukin‐8 [IL‐8], interleukin‐12 [IL‐12], TNF, and IFN) and downregulates the expression of CD86, which inhibits antigen presentation [87]. Shea et al. [88] devised a smart way to manipulate macrophages using viral vectors to reduce leukocyte infiltration during abdominal fat implantation by delivering IL‐10 gene vectors via poly (propionyl‐glycolide) scaffolds (PLGs), which corresponded to an increase in macrophage IL‐10 expression and a decrease in inflammatory cytokine secretion by dendritic cells and T cells (Figure 7A). Similarly, Jain et al. [89] observed similar positive results when they treated arthritic rats with alginate‐based nanoparticles loaded with IL‐10 plasmid DNA, and their experimental results showed that the loaded particles achieved macrophage polarization toward a reparative phenotype as compared with the unloaded or nontargeted particles (Figure 7B), which demonstrated the role of IL‐10 as a phenotypic modulator of the giant snorting cells. Based on these long‐lasting immunomodulatory properties of IL‐10, recent studies have shown its excellent ability to modulate immune responses, and therefore, it has recently been proposed that IL‐10 can be used in the clinic alone or in combination with other types of biomolecules to modulate immune responses.

In addition to IL‐10, other cytokines, such as IL‐4, have been investigated for immunomodulatory therapies, and it has been shown that IL‐4 stimulates macrophages to shift from a pro‐inflammatory to a pro‐repairing phenotype and promotes the proliferation of anti‐inflammatory cells [90]. Kumar et al. [91] developed a self‐assembling peptide/heparin (SAP/Hep) hydrogel and designed a biphasic release pattern of cytokines from IL‐4 and MCP‐1, a chemokine released from activated monocytes that plays an important role in proinflammatory stimulation through the production of reactive oxygen/reactive nitrogen. In this study, the active interaction between the fibrous hydrogel and macrophages, as well as the biphasic release pattern of cytokines, led to the activation of THP‐1 monocytes and macrophages, which enhanced the anti‐inflammatory phenotype polarization. The potential of IL‐4 in modulating inflammatory responses and pro‐repairing phenotypic macrophages has been further demonstrated in other tissues such as skeletal muscle, where gold nanoparticles containing IL‐4 led to an increase in the replacement of activated macrophage phenotypes, Importantly, treatment with dissolved IL‐4 did not lead to any changes in macrophage polarization or improvement in muscle function compared with controls, highlighting the benefits of biomaterials as delivering agents benefits of biomaterials as delivery agents (Figure 7C) [92]. Another effort to load IL‐4 onto nanoscale chitosan coatings on polypropylene surgical mesh promoted polarization of the replacement‐activated macrophage phenotype in vitro, suggesting an increased replacement‐activated macrophage/classically‐activated macrophage ratio and reduced fibrous envelope thickness after envelope‐coated implantation compared with uncoated controls, with the potential for improved integration (Figure 7D) [93].

The delivery of signaling molecules in hydrogels increases opportunities for improved medical device integration and increased therapeutic benefits. However, some cytokines encounter difficulties during delivery due to the need to achieve biologically relevant localized concentrations. Therefore, further optimization of the stable controlled release mechanisms of the molecules is needed to improve the efficacy of each active substance. At the same time, such delivery must also be considered in the context of complex signaling networks, taking into account their effects on the hydrogel itself.

##### 2.3.2.3 Delivery of Immune Cells

Transfusion of different cell types with multiple differentiation potentials has also proven to be a promising strategy for tissue regeneration. For example, exogenous allogeneic islet cell infusion has recently been used clinically in the treatment of type I diabetes mellitus. However, this process requires the use of immunosuppressive drugs to prevent the activation of an overactive immune system in the patient's body. Similarly, giant thumb cells and other pluripotent stem cells, such as mesenchymal stem cells (MSCs), adipose stem cells (ASCs), and human umbilical cord blood serum (hUCBs), have shown significant regenerative potential in the treatment of chronic trauma [94, 95, 96, 97]. Bliley et al. [98] found that the ratio of messenger RNA (mRNA) expression of type II to type I collagen, vascular density, and collagen deposition was improved after transplantation of ASCs into burn wounds. In addition, it has been shown that implantation of human amniotic epithelial cells (AMEs) also exhibits high implantation rates and leads to accelerated wound healing [99]. However, direct use of cells for therapy is limited by various risk factors, including tumor formation, thrombosis, and unwanted immune responses [100]. Therefore, delivering therapeutic cells through hydrogels to maintain their activity and reduce unwanted immune responses becomes an effective strategy.

Firstly, macrophages have become a hotspot for research on immune cells delivered by biomaterials due to their key role in immunomodulation. For example, Hu et al. [94] delivered more than physiological amounts of inactivated macrophages to a mouse skin wound model using Pullulan collagen hydrogel, and the transplanted cells survived for at least 7 days in vivo and migrated to the middle and lower dermis and acquired a mixed M1/M2 phenotype, accelerating wound healing and angiogenesis. Interestingly, the authors found that macrophages from diabetic mice also accelerated diabetic wound healing, and even transplantation of human diabetic monocytes accelerated wound healing in immunodeficient mice, although the cell survival of the transplanted cells or their incorporation into mouse tissues has not been assessed. Despite these encouraging results, the efficacy of monocyte/macrophage‐based therapies is still limited because the high plasticity of exogenously administered macrophages leads them to present microenvironmental stimulus‐induced phenotypes at the site of injury, and it is not possible to control their phenotypic changes [101, 102]. In addition, MSCs are a widely studied cell source for regenerative medicine with broad immunomodulatory capabilities that can provide adjuvant therapy by modulating adaptive and innate immunity through paracrine mechanisms [103]. Although it is initially studied that they can differentiate into various mesenchymal cell types (such as bone and cartilage), they are now more widely used due to their immunomodulatory properties. Thus, MSC therapy has the potential to modulate macrophage phenotype to enhance tissue repair; however, MSC transplantation suffers from low in vivo survival and cell retention [104, 105, 106]. In addition, ongoing studies have concurrently shown that transplantation of bone marrow MSCs may cause adhesion of microorganisms such as Staphylococcus aureus and colony formation of *S. aureus* planktonic or biofilm [107]. Therefore, hydrogels have been developed to protect MSCs in many applications, and encapsulation of MSCs in suitable hydrogels can effectively impede colony formation of planktonic *S. aureus* while maintaining the pluripotency of MSCs [108]. For example, the use of hydrogel‐encapsulated MSCs has been shown to reduce their fiber encapsulation, and the ability of this effect decreases with increasing cell differentiation [109]. Clark et al. [110] investigated how to deliver MSCs and modulate their function by different integrin‐modified hydrogels. They modified PEG hydrogels with peptides that could bind to different integrins expressed by MSCs, such as GFOGER and RGD peptides. The former was derived from type I collagen, with binding specificity for α2β1 integrin, and the latter could be found in different ECM proteins, such as fibronectin, with binding specificity for αγβ3, αγβ1, and α5β1 integrins. The results showed that GFOGER hydrogels prolonged the survival time of MSCs, upregulated many genes and cytokines associated with inflammation, and enhanced their ability to stimulate bone repair in a mouse segmental defect model. Inflammatory cytokines promote MSCs to increase their immunomodulatory properties in a wound healing environment [111]. In addition, a self‐healing hydrogel loaded with adipose‐derived MSCs exosomes (ADSCs‐exo) has been used for the treatment of damaged skin, which was synthesized by a Schiff base chemical reaction between oxidized HA and poly‐ε‐L‐lysine in the presence of Pluronic F127. The hybrid hydrogels loaded with AMSCs‐exo were easy to inject for significantly promoting the healing of diabetic skin [112]. Similarly, another injectable hydrogel was synthesized through the interaction of hyperbranched acrylic polyethylene glycols (HP‐PEGs) and sulfated HA for effective treatment of ADSCs. In addition to outstanding mechanical properties and antifouling properties, the hydrogel loaded with ADSCs showed good regenerative ability for the potential treatment of diabetic wounds [113].

## 3 Multifunctional Hydrogel Dressing and Preclinical Formulations for Wound Healing

Hydrogel dressing is not only able to effectively absorb blood and exudate from the wound, but also able to provide a long‐lasting wet environment for the wound, while delivering drugs or growth factors to the wound site through the continuous release of encapsulated therapeutic agents, thus accelerating wound healing. Therefore, hydrogels show unique advantages in skin wound repair and have become an important wound dressing material. Based on their excellent properties, various types of hydrogels have been designed and developed and have been widely used in clinical treatments [114, 115, 116, 117, 118, 119, 120, 121, 122]. Different types of skin wounds have different shapes (size, thickness, etc.) and clinical manifestations (necrosis, putrefaction, etc.), and the ideal hydrogel wound dressing should have the functions of rapidly forming an anti‐infective barrier, promoting rapid blood coagulation, absorbing wound exudate, blocking nerve endings to reduce pain, and providing nutrients to promote tissue regeneration, etc. So, a variety of hydrogel dressings have been developed in accordance with the needs of repair (Table 1).

**TABLE 1:** Function‐based summary of multifunctional hydrogel dressings.

Function	Mechanism	Representative systems	Models	Outcomes	Limitations	Ref.
Antibacterial	Membrane disruption; ion‐mediated ROS; photothermal sterilization; antifouling	Chitosan/cationic polymers; Ag/Au/Zn/Cu NPs; MoS_2_ + NIR; PEG/zwitterionic	In vitro (MRSA*, E. coli*); diabetic mouse; porcine burns	Reduced bacterial burden; faster closure	Ion/cargo cytotoxicity; resistance if antibiotics used alone	[123, 124, 125, 126, 127, 128, 129, 130]
Hemostatic adhesion	Schiff base (aldehyde–amine) and catechol‐mediated wet adhesion	Catechol‐grafted HA/gelatin; aldehyde‐chitosan; PEG‐catechol	Rodent and porcine wounds	Rapid hemostasis; strong wet adhesion	Reactivity of carbonyls; long‐term stability	[131, 132, 133, 134]
Self‐healing	Dynamic covalent (Schiff, acylhydrazone, disulfide, boronate); supramolecular	Oxidized polysaccharide + amine; boronate esters; host–guest gels	Joint‐overlying wounds; injectability models	Barrier restoration under strain; painless removal	Strength–dynamics trade‐off	[135, 136, 137, 138, 139]
Antioxidant	ROS scavenging; anti‐inflammatory signaling	Polyphenol‐loaded hydrogels	Diabetic/infected rodent wounds	Lower ROS and cytokines; improved closure	Cargo stability; release kinetics	[140, 141, 142, 143, 144, 145]
Drug/GF delivery	Staged or zero‐order release	EGF/FGF/KGF gels; NO‐donor fibers	Rodents; primates for GFs	Accelerated epithelialization; less scarring	Burst release risk; CMC complexity	[146, 147, 148, 149, 150, 151]
Stimuli‐responsive	Triggered by external/internal signals (temperature/pH/NIR/ enzymes) to alter structure (e.g., swelling, degradation, drug release)	PNIPAM‐based thermogels; carboxyl/amino‐functionalized hydrogel; MoS_2_/GO photothermal composites; matrix metallo‐proteinase‐cleavable peptides	In vitro (stimulus‐induced release kinetics); in vivo (diabetic wounds with pH/ROS microenvironment)	On‐demand dosing; real‐time monitoring	Trigger safety; device robustness; hysteresis in response	[152, 153, 154, 155, 156, 157, 158, 159, 160, 161, 162]
Wearable	Mechanical flexibility, skin conformal adhesion, and integration with sensing/actuating modules	Double‐network hydrogels; catechol/chitosan‐based wet adhesives; conductive hydrogels	In vivo (dynamic wound sites); human trials (long‐term wearability); mechanical testing (tensile strain and cyclic durability)	Comfortable fit under body movement; real‐time monitoring; reusable/peelable without trauma	Signal drift in biofluid‐rich environments; limited breathability for extended wear	[163, 164, 165, 166, 167, 168, 169, 170, 171]

Abbreviations: GF, growth factor; CMC, chemistry, manufacturing, and controls; NO, nitric oxide.

### 3.1 Antibacterial Hydrogels

Bacterial infection is one of the most common complications during the wound healing process. When a wound is infected, bacteria can cause a sustained inflammatory response in the infected area of the wound, prolonging the wound healing cycle and even causing complications such as sepsis. Currently, the clinical use of antibiotics is effective in controlling wound infections caused by common bacteria, but due to the misuse of antibiotics, the emergence of bacterial resistance poses a great difficulty in clinical treatment. For skin infections caused by drug‐resistant bacteria, antibiotics are not effective in controlling the infection, so it is imperative to find more effective antimicrobial strategies that do not cause the development of bacterial resistance.

**FIGURE 8:**
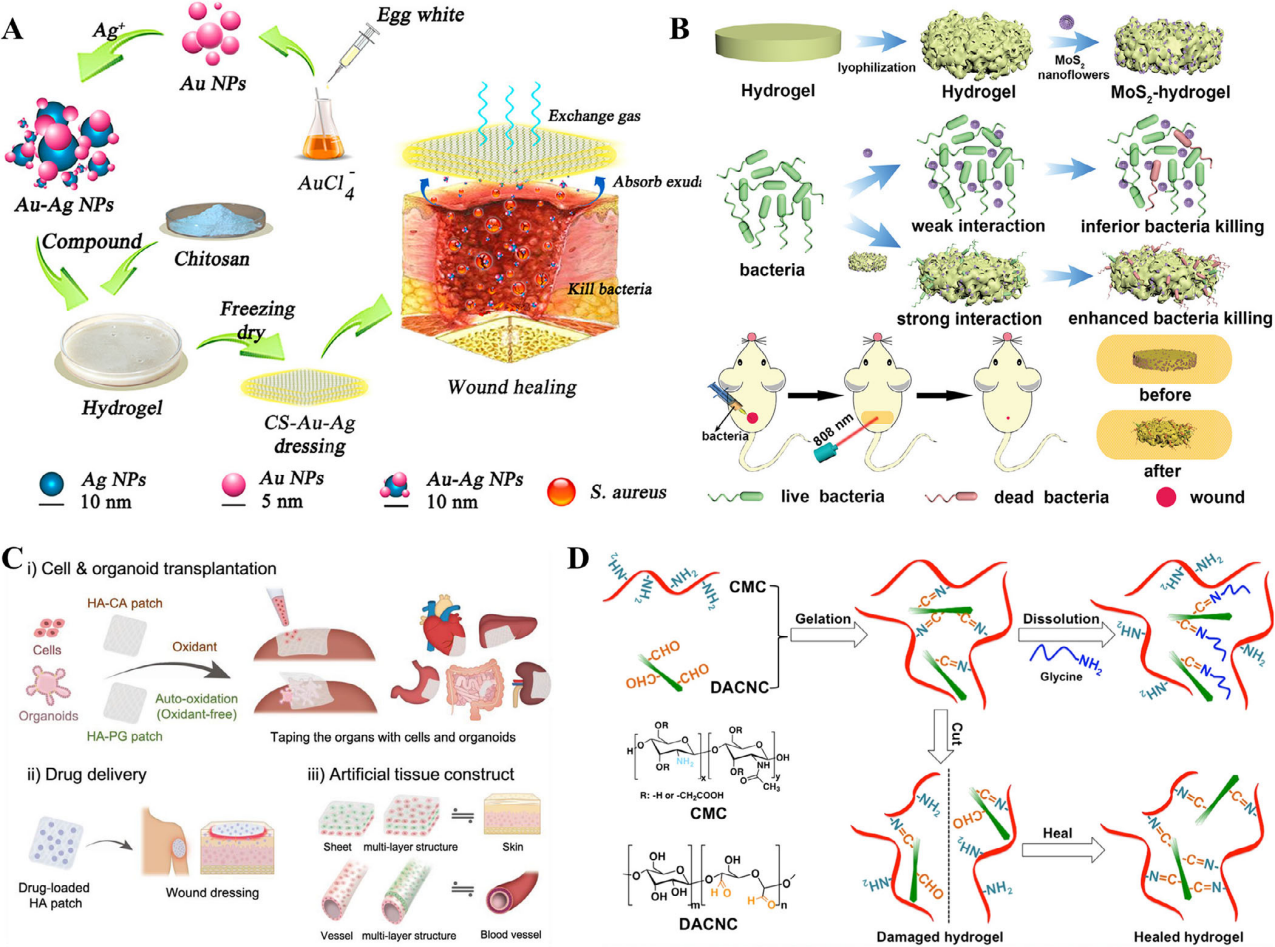
Multifunctional hydrogel dressings for wound healing. (A) Conceptual illustration of the preparation for CS–Au–Ag and use as a wound dressing. *Source*: Reproduced with permission from [125]. Copyright 2017, ACS Publications. (B) Schematic illustration of the synthesis of MoS_2_‐hydrogel and the MoS_2_‐hydrogel used for bacteria clearance and the treatment of wound infection. *Source*: Reproduced with permission from [126]. Copyright 2019, Wiley. (C) Various applications of oxidant‐dependent HA‐CA patch and oxidant‐free HA‐PG patch; (i) transplantation of single cells and massive cell clusters (organoids) to various types of organs, (ii) sustained drug delivery system for wound healing, and (iii) construction of multilayered tissue structures such as skin and blood vessels. *Source*: Reproduced with permission from [134]. Copyright 2019, Wiley. (D) Schematic illustration for gelation and on‐demand dissolution of CMC/DACNC hydrogel. Amine groups of CMC reacted with aldehyde groups of DACNC to form reversible and dynamic Schiff‐base linkages. After adding glycine into the dynamic CMC/DACNC hydrogel, the amine groups of glycine replaced the amine groups of CMC and reacted with the aldehyde groups of DACNC to form new Schiff‐base linkages, and finally, the hydrogel network was broken. CMC: carboxymethyl chitosan; DACNC: dialdehyde‐modified cellulose nanocrystal. *Source*: Reproduced with permission from [139]. Copyright 2018, ACS Publications.

Inorganic metal nanoparticles such as silver (Ag), gold (Au), zinc (Zn), and copper (Cu) have been widely used in antimicrobial treatments of skin [123, 124]. Although their biosafety issues and potential risks have not been effectively addressed, metal nanoparticles are still one of the most commonly used antimicrobial agents besides antibiotics due to their high antimicrobial efficacy. For example, Li et al. [125] prepared chitosan hydrogels doped with Ag‐Au composite nanoparticles showing good antimicrobial activity (Figure 8A). Subsequently, other metal nanoparticles have been introduced to the study of antimicrobial therapy. For example, Qu et al. [126] doped MoS2 nano‐enzymes (which can catalyze the conversion of H_2_O_2_ to O_2_) into poly‐isopropylacrylamide hydrogels, which can effectively remove bacteria, control infections, and inhibit inflammatory reactions through the synergistic sterilization of near‐infrared photo‐thermal effect and reactive oxygen species (ROS, Figure 8B). Hydrogel with a cationic surface can destroy the bacterial cell membrane through the positive charge on its surface and the negative charge on the bacterial surface to exert a bactericidal effect [127, 128, 129]. As the only natural cationic polysaccharide, chitosan has excellent antimicrobial activity in cooperation with natural or synthetic polymers such as gelatin, dextran, lignin, carbon dots, or strong cations such as quaternary ammonium salts, polylysine, etc., to further enhance the antimicrobial effect [130].

### 3.2 Adhesive Hemostatic Hydrogels

Skin hemostatic adhesive is a repair material that can be firmly bonded to damaged skin tissues, which can be bonded to close the wound to stop bleeding and prevent the loss of tissue fluid, shrinking the wound to promote healing [131]. Outstanding hemostatic dressing in the hydrogel on the tissue adhesion assistance, not only can stay in the wound site for a longer period, to play the effect of physical closure, but also enriches the hemostatic factor in the wound area, accelerates hemostasis [132]. Hydrogel‐tissue adhesion is mainly divided into two modes of action: physical and chemical. Physical action is mainly based on the physical interactions between hydrogel functional groups and tissue surface functional groups, such as hydrophobic interactions, electrostatic interactions, host–guest complexation, etc., while chemical action is the formation of covalent bonding between the hydrogel surface functional groups and the tissue surface functional groups by chemical reaction, which makes the hydrogel adhesion to the tissue stronger. For example, the aldehyde group of the hydrogel side chain can react with the amino group on the surface of the tissue to form an amide bond, which produces a stronger tissue adhesion effect [133]. Inspired by the strong adhesion of marine mussels to wet surfaces, various skin adhesives based on catechol moieties have been developed. Shin et al. [134] prepared catechol/catechol‐grafted HA hydrogel patches with excellent adhesive hemostatic effect on skin wounds, and the synthesized portable wound patches containing stem cells can stay in the traumatized area for a long time and play a therapeutic role (Figure 8C).

### 3.3 Self‐Healing Hydrogels

In the case of hydrogels, in addition to their rapid hemostatic function, the physical barrier they form is also effective in preventing external contamination. When a wound exists on a skin surface that is often stretched, such as a joint area, the hydrogel is often prone to rupture under the influence of the external tension generated by the skin tissue activity, leading to the loss of the hydrogel's physicochemical properties and increasing the risk of wound infection [135]. Therefore, self‐healing hydrogels have been applied to the field of skin dressings. The healing mechanism of self‐healing hydrogel is mainly divided into two ways: physical self‐healing and chemical self‐healing. Physical self‐healing hydrogels are dynamically reconstructed by noncovalent dynamic interactions between polymer chains (e.g., hydrophobic interactions, host–guest interactions, and hydrogen bonding) to realize the network. Chemical self‐healing hydrogels are constructed by dynamic covalent bonding between polymer chains (e.g., phenylborate, disulfide bonding, reversible free radical polymerization, acylhydrazone bonding, and amide bonding) to build a dynamic hydrogel network [136, 137, 138], where amide bonding, mainly amino‐ and aldehyde‐based reactions, is most used in the preparation of skin‐healing hydrogels. For example, Huang et al. [139] used the Schiff base reaction between the amino group on the chain segment of soluble carboxymethyl chitosan and the aldehyde group on oxidized cellulose to prepare an injectable self‐healing, amino acid‐dissociating, painless peeling hydrogel for deep burn wound repair (Figure 8D).

### 3.4 Antioxidant Hydrogels

**FIGURE 9:**
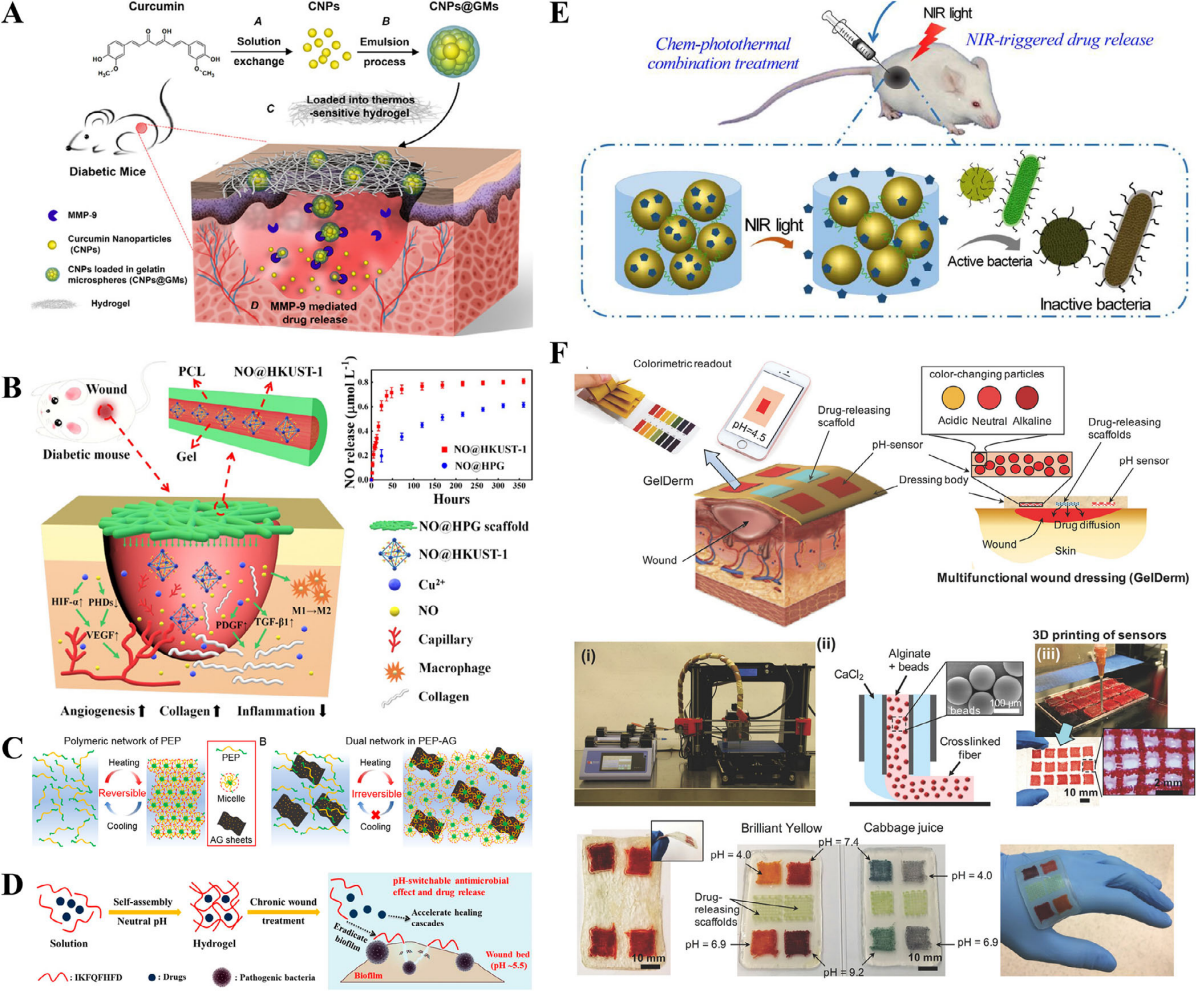
Schematic illustrations of key drug delivery and wound management technologies. (A) Schematic representations of CNPs@GMs/hydrogel preparation and the process of drug release at the wound bed in diabetic mice. CNPs: cur nanoparticles; GMs: gelatin microspheres. *Source*: Reproduced with permission from [145]. Copyright 2018, ACS Publications. (B) Schematic illustration of an electrospun film loaded with NO gas molecules promoting vascular regeneration in damaged skin tissue. NO@HPG: NO@HKUST‐1/PCL/Gel. *Source*: Reproduced with permission from [151]. Copyright 2020, ACS Publications. (C) Schematic structures in the PEP hydrogel and the PEP‐AG composite hydrogel. PEP: poly (*N*‐isopropylacrylamide_166_‐*co*‐*n*‐butyl acrylate_9_)‐poly (ethylene glycol)‐poly (*N*‐isopropylacrylamide_166_‐*co*‐*n*‐butyl acrylate_9_) copolymer (P(NIPAM_166_‐*co*‐nBA_9_)‐PEG‐P(NIPAM_166_‐*co*‐nBA_9_); AG: silver‐nanoparticles‐decorated reduced graphene oxide nanosheets. *Source*: Reproduced with permission from [158]. Copyright 2019, ACS Publications. (D) Conceptual illustration of pH‐switchable antimicrobial IKFQFHFD‐based nanofiber networks for biofilm eradication and rescuing stalled healing in chronic wounds. IKFQFHFD: an octapeptide of Ac‐Leu‐Lys‐Phe‐Gln‐Phe‐His‐Phe‐Asp‐NH_2_. *Source*: Reproduced with permission from [160]. Copyright 2019, ACS Publications. (E) Schematics of the NIR light irradiation‐triggered Cip release from Gel‐Cip for bacterial inactivation. Cip: ciprofloxacin hydrochloride. *Source*: Reproduced with permission from [162]. Copyright 2019, Elsevier. (F) Schematic illustration of an advanced multifunctional hydrogel‐based dressing (GelDerm) for monitoring and management of wounds. This dressing is capable of colorimetric measurement of pH as an indicator of bacterial infection and releasing antibiotics to wound sites. *Source*: Reproduced with permission from [170]. Copyright 2017, Wiley.

During the inflammatory phase, immune cells are recruited to the wound area and secrete various cytokines to regulate the wound healing process, while phagocytosing debris and foreign bodies to maintain the homeostasis of the wound microenvironment. Appropriate inflammation is essential for wound repair, but when external factors interfere with the wound healing process, the secretion of inflammatory factors is disrupted, and macrophage polarization fails, at which point the local microenvironment becomes dysregulated, leading to a prolonged inflammatory phase of the wound. Excessive inflammation leads to high oxidative stress and a dramatic increase in reactive oxygen species (ROS, mainly superoxide anion, hydroxyl radical, hydrogen peroxide, etc.), which triggers a cascade of reactions (e.g., liposomal, DNA/protein peroxidation, etc.) to damage cells and affect the healing process [140]. Therefore, hydrogels with antioxidant properties can modulate the inflammatory response and significantly improve the state of wound healing [141]. Among the commonly used antioxidants, natural polyphenols are widely used because of their excellent antioxidant stability and ease of storage. Common natural polyphenols such as polyphenols, resveratrol, anthocyanins, curcumin, and flavonoids are often incorporated into hydrogels to exert antioxidant effects to promote wound healing [142, 143, 144]. For example, Qu et al. [145] prepared polyphenol micelles to enhance the delivery efficiency of drug‐carrying drugs by encapsulating them in block copolymer hydrogels, and to maintain the local antioxidant effect in vivo with long‐lasting, slow release (Figure 9A).

### 3.5 Drug‐Delivery Hydrogels

The porous structure of hydrogel makes it suitable for loading and slow release of various biofunctional factors. Compared with direct drug delivery, hydrogel‐assisted delivery can control the drug dosage and maintain the stabilization of biofactor activity to achieve the effect of precise and slow release [146, 147]. Cytokines are an important class of biologically active proteins that play an important role in regulating cell function and maintaining tissue homeostasis. Numerous studies have shown that topical delivery of epidermal growth factor (EGF), fibroblast growth factor (FGF), keratinocyte growth factor (KGF), and nerve growth factor (NGF) via hydrogel can promote skin wound healing in primate models, and has been used to a certain extent in clinical practice [148, 149]. Shi et al. [150] used hydrogel microspheres loaded with EGF and then encapsulated them into FGF‐containing block hydrogels, which resulted in the secondary release of two growth factors, achieving rapid wound healing and avoiding keloidal scarring due to overexpression of FGF at the late stage of wound healing. Gas molecules such as NO have a vasodilatory effect, which promotes blood circulation and accelerates local metabolism during the wound healing process, while NO also increases the mechanical strength of newborn skin by promoting collagen deposition. Xu et al. [151] used the core‐shell electrospinning technology, the internal encapsulation of NO gas molecular retardant, and the external wrapped by polycaprolactone (PCL). This microfilament film can significantly prolong the period of NO gas retardation. This film covers the skin wound area, which can significantly accelerate the speed of wound closure (Figure 9B).

Since stem cells not only proliferate and differentiate into a variety of cells for tissue repair during the tissue healing process, but also secrete a variety of cytokines and chemokines to regulate the wound healing process, selective delivery of exogenous stem cells has a great advantage in promoting wound healing. Among various exogenous stem cells, ADSCs are the main source of cell therapy to secrete cytokines that favor wound repair and stimulate vascular and epithelial regeneration, thus promoting the functional recovery of damaged tissues [152, 153]. Eke et al. [154] achieved in situ ADSCs loading by light‐curing gelatin and hyaluronic acid, with the highest in vivo survival of stem cells, the highest amount of neoplastic skin tissue vascular regeneration, and the fastest rate of repair in the hydrogel‐delivered stem cell group, relative to the blank hydrogel group and the direct delivery of ADSCs group.

### 3.6 Stimuli‐Responsive Hydrogels

Stimulus‐responsive hydrogels are hydrogels that change their physicochemical properties in response to perturbations in the external environment (e.g., temperature, pH, light, glucose) [155, 156]. The low critical solubilization temperature (LCST) of thermosensitive hydrogels for wound dressings is the physiological temperature of 37°C. Therefore, the injectable low‐temperature flowable hydrogel precursor solution can be rapidly transformed to a nonflowable gel state at physiological temperature on the body surface. N‐isopropylacrylamide (NIPAM) with a LCST of about 32°C, which is similar to the physiological temperature of the body [157]. For example, Yan et al. [158] modified NIPAM into a block copolymer and then added Ag‐reduced graphene nanosheets to it to confer antimicrobial properties, and the hydrogel could undergo a reversible sol–gel transition as the temperature changed, facilitating hydrogel skin wound coating and painless removal (Figure 9C).

The pH range of normal skin is 4.0–6.0, and during the hemostatic and inflammatory phases of early acute repair of skin injuries, pH fluctuates due to imbalances in local tissue homeostasis, so pH‐responsive hydrogels can be used for acute wound drug delivery therapy [159]. For example, Li et al. [160] synthesized peptides that can de‐self‐assemble and solubilize with pH change, and under acidic environment, the supramolecular peptide structure de‐assembles, and slow‐release antimicrobial peptide molecules disrupt bacterial cell membranes and inhibit chronic wound infections (Figure 9D). Photo‐responsive hydrogels have the advantages of a fast response rate and easy control of gel formation [161]. The most common photo‐responsive hydrogel design strategies for skin dressings are infrared light irradiation to warm the hydrogel, kill bacteria, or facilitate the release of internally encapsulated drugs. Wu et al. [162] prepared chitosan hydrogels loaded with ciprofloxacin antibiotics and polydopamine nanoparticles, and the antibiotics were not exuded inside the hydrogel under room temperature, whereas under near‐infrared light conditions, the temperature of the hydrogel increased, the pores increased, and the antibiotics began to be released slowly outward to inhibit the wound bacterial growth (Figure 9E).

### 3.7 Smart Wearable Hydrogels

From single‐function skin wound dressings to multifunctional dressings in recent years, researchers have continued to explore the treatment strategy of disposable wound delivery, but skin wound healing is a complex dynamic process [163, 164, 165, 166, 167, 168]. If real‐time monitoring of wound parameter indicators and timely adjustment of the treatment plan can be achieved, precise treatment can be realized to achieve a more perfect repair effect [169]. With the rapid development of wearable devices, a range of smart wearable hydrogels has been developed for wound repair. For example, Akbari et al. [170] wrapped pH‐responsive color‐changing mesoporous resin microspheres with sodium alginate and prepared hydrogel skin wound dressings containing sensors by 3D printing, which can determine the bacterial infection status from the color change and adjust the antibiotic dose in real‐time. Digital remote diagnosis and treatment can also be realized when connected to an image acquisition device (Figure 9F). Pang et al. [171] developed a two‐layer hydrogel sensor with an upper layer of polydimethylsiloxane integrated with a temperature sensor and UV light‐emitting diode, and a lower layer of UV‐sensitive hydrogel encapsulating antibiotics. It can monitor the wound for early infection through the integrated sensors, and then use the upper UV radiation to the lower hydrogel to regulate the release dose of antibiotics, and achieve on‐demand drug delivery and infection control by real‐time temperature tracking of the wound.

### 3.8 Preclinical and Clinical Hydrogels for Wound Dressings

Translation from bench to bedside has been supported by a steady accumulation of preclinical and clinical evidence. Rodent models remain essential for mechanistic dissection, whereas rabbits are often used for vascular and cartilage endpoints, and porcine skin models provide the closest physiological surrogate for human epidermal/dermal structure. Clinically, alginate‐, HA‐, PEG‐, and fibrin‐based formulations have been evaluated for chronic wounds (DFU, pressure ulcers) and burns. For example, the clinical trials have been conducted for the treatment of DFU using alginate dressings or commercial products (e.g., Sorbalgon, Hydrofiber). Similarly, there are many injectable hyaluronic acid (HA)‐based hydrogel dressings in clinical trials or have already been commercialized (e.g., REVITACARE, RETILAN) for wound healing [172, 173, 174]. They have shown faster closure, reduced infection rates, and improved scar quality, albeit with device‐specific variability and persistent challenges in manufacturing control and standardization [175, 176, 177, 178, 179]. A concise selection is summarized below (Table 2).

**TABLE 2:** Selected preclinical and clinical studies of hydrogel wound dressings.

Hydrogel system	Route	Mechanism	Model	Outcomes	Limitations	Ref.
Alginate dressings	Topical	Moist microenvironment; exudate control	Porcine partial‐thickness burns; clinical DFU	Faster closure; reduced infection/exudate	Limited mechanics; maceration if overocclusive	[172, 174, 175]
HA injectable gels	Injectable/in situ gelling	ECM mimicry; promigratory, proangiogenic	Rat full‐thickness; pilot clinical burns	Enhanced re‐epithelialization; improved scars	Rapid enzymatic degradation; requires crosslink tuning	[176, 177, 178]
PEG‐GF composites (EGF/FGF)	Topical/injectable	Sequential GF release	Mouse excision; primates	Granulation ↑; closure ↑; scarring ↓	Cost; burst release if poorly engineered	[148, 149, 150]
NO‐releasing fiber films	Topical	Vasodilation; perfusion; collagen deposition	Rodent full‐thickness	Significantly faster closure	NO donor stability and dose control	[151]
MSC/ADSC hydrogel delivery	Injectable/patch	Cell retention; paracrine angiogenesis and immunomodulation	Diabetic rat ulcers; early‐phase clinical exploration	Vascular density ↑; closure rate ↑	Cell viability; immune risk; CMC reproducibility	[152, 153, 154]

Based on the above research progress and discussions, it is concluded that rapid healing of any type of wound is particularly important, as skin wounds affect all aspects of life in one way or another. However, the underlying pathologic causes of delayed or nonhealing wounds vary, making wound care complex and challenging. In addition, a range of properties, including degradation time, degradation products, and their effects (short/long‐term effects on the human body), are underindicated and understudied, which we speculate is related to the complex composition and properties of tissue wounds. In addition, the removability of hydrogel dressings cannot be ignored, as it is directly related to their replacement during use and removal at the end of use, and further relates to the therapeutic effect and the safety and comfort of patient use. Therefore, reasonable removal strategies and removal performance evaluation need to be included in the research.

While substantial research has delved into utilizing hydrogels for healing skin wounds, effectively treating chronic and deep wounds remains a persistent challenge. To achieve this objective, it is necessary to discover more multifunctional hydrogel‐based wound dressing materials with the following features: (1) Advanced mechanical performances of hydrogels to withstand the dynamic nature of wound environments. (2) Smart hydrogel dressings to dynamically respond to changes in the wound environment and adapt properties based on specific healing stages. (3) Controlled drug delivery capability to load and release therapeutic agents to promote healing and prevent infections. (4) Suitable degradation profiles to address concerns about their durability and possible removal methods. (5) Customized shape and size according to the wound needs, like skin type, and wound characteristics, to improve the effectiveness and reduce side effects. (6) Injectable formulation requirement to facilitate the ease of operation, compatibility with the minimally invasive operations, ability to conform to deep irregular defects, and avoidance of damage to fragile tissues. Consequently, it is necessary and urgent for scientists and clinicians to gain a deeper understanding of the causes and differences in the formation of various types of tissue wounds at the molecular and cellular levels, to develop more effective wound management products that can promote healing and re‐establish function through tissue regeneration rather than simple surgical repair.

## 4 Multifunctional Hydrogels in Tissue Engineering and Regenerative Medicine

While hydrogels have been extensively studied in the context of cutaneous wound healing, their multifunctional properties also make them highly attractive for applications across diverse fields of tissue engineering and regenerative medicine. By tailoring their chemical composition, structural design, and biological functionalities, hydrogels can be adapted to meet the unique regenerative requirements of various tissues. The following subsections simply summarize recent progress in typical application examples, including bone and cartilage regeneration, cardiac repair, neural tissue regeneration, corneal tissue repair, visceral organ repair, and gastrointestinal tissue regeneration.

### 4.1 Bone and Cartilage Tissue Regeneration

**FIGURE 10:**
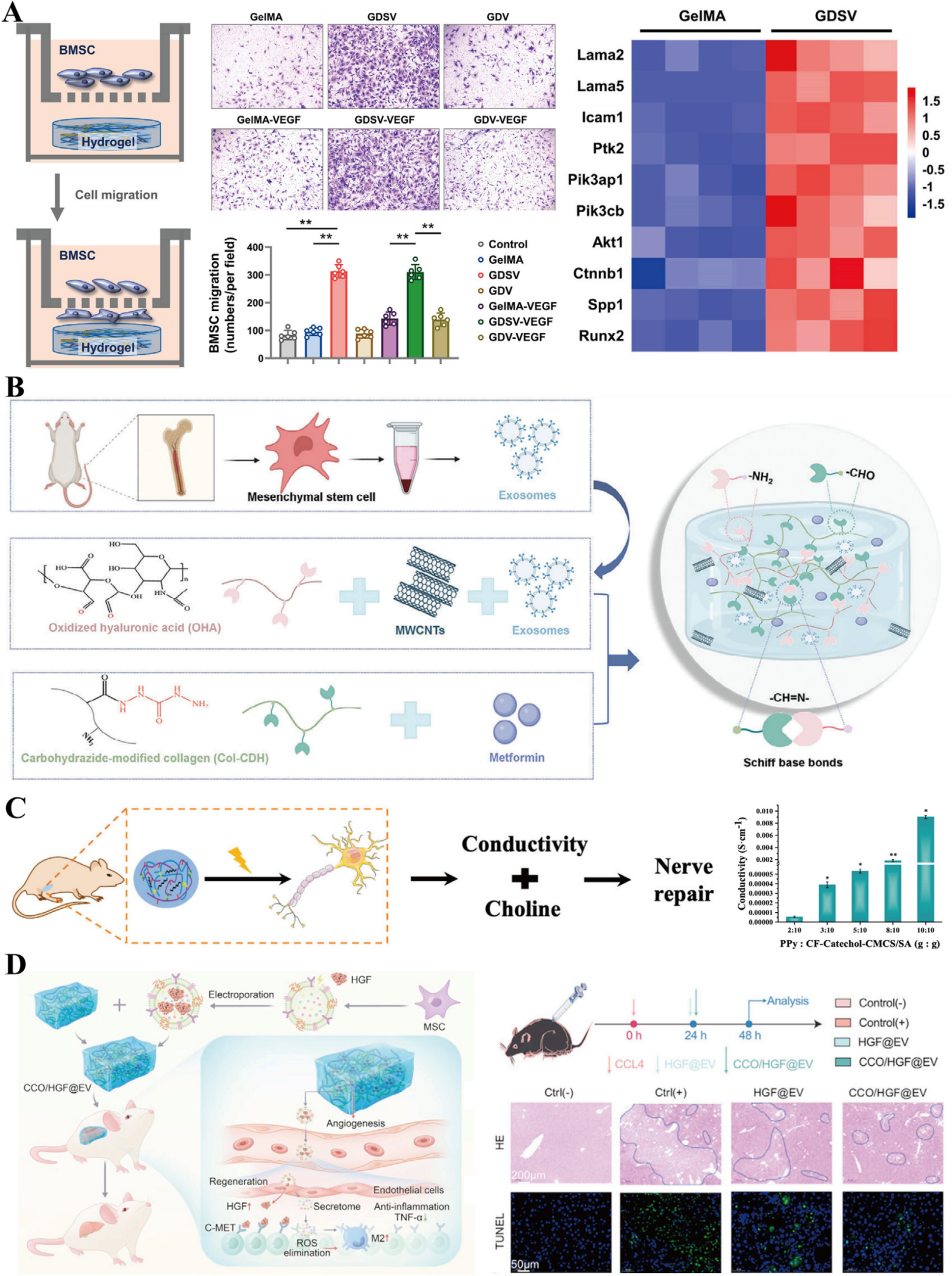
Multifunctional hydrogels in tissue engineering and regenerative medicine. (A) GDSV hydrogel for promoting BMSCs homing and heat map of DEGs related to cell adhesion and osteogenesis in the GDSV group vs. the GelMA group. GDSV: GelMA/DNA/Apt19S/AptV; Apt19S: an aptamer proved to label multipotent stem cells such as MSCs; AptV: the VEGF aptamer. *Source*: Reproduced with permission from [183]. Copyright 2024, Elsevier. (B) Schematic diagram of exosome extraction and hydrogel synthesis. Exosomes are extracted from rat bone marrow mesenchymal stem cells, and the hydrogel system is constructed by Schiff base reaction based on oxidized hyaluronic acid (OHA) and carbohydrazide‐modified collagen (Col‐CDH). *Source*: Reproduced with permission from [187]. Copyright 2025, Wiley. (C) Schematic diagram of nerve repair with hydrogel and the conductivity of CF‐Catechol‐CMCS/SA/PPy hydrogels with varied mass ratio of PPy and CF‐Catechol‐CMCS/SA. CF: choline functionalized; CMCS: carboxymethyl chitosan; SA: sodium alginate; PPy: polypyrrole. *Source*: Reproduced with permission from [191]. Copyright 2024, Elsevier. (D) Synthesis pathway and therapeutic mechanism of CCO/HGF@EV and treatment efficiency of acute liver injury by CCO/HGF@EV in vivo. CCO: polysaccharide‐based hydrogels; HGF: hepatocyte growth factor; EV: exosomal vesicles. *Source*: Reproduced with permission from [193]. Copyright 2025, Elsevier.

Skeletal defects and degenerative diseases such as osteoarthritis remain major challenges due to the limited self‐healing capacity of bone and cartilage [180]. Hydrogels provide a hydrated three‐dimensional network that mimics native ECM and facilitates the delivery of osteogenic or chondrogenic factors [181, 182, 183, 184, 185]. Recent studies have developed a kind of injectable hydrogel incorporating bone morphogenetic proteins (BMP‐2, BMP‐7), transforming growth factor‐beta (TGF‐β3), or MSC‐derived exosomes, which significantly enhance osteogenesis and chondrogenesis. For example, Li et al. [186] developed a GelMA/DNA composite hydrogel for bone regeneration. By utilizing stress relaxation and aptamer recruitment of BMSCs, as well as continuous release of VEGF, it accelerated the vascularized bone regeneration by activating the FAK/PI3K/Akt/β‐Catenin pathway (Figure 10A). Furthermore, the hybrid hydrogels reinforced with bioactive ceramics or nanomaterials further improved mechanical stability and support long‐term defect filling. Yang et al. [187] fabricated a ceramic scaffold loaded with β‐TCP through 3D bioprinting. They regulated the immune‐bone formation sequence by sequentially releasing IL‐4 and alendronate, promoting the regeneration of osteoporotic bone defects and inhibiting bone resorption. Moreover, self‐healing and adhesive hydrogels enabled the minimally invasive administration while maintaining strong integration with the surrounding tissues, an essential feature for irregular bone defects and articular cartilage repair. Jiang et al. [188] prepared a biphasic hydrogel scaffold with self‐healing properties and a continuous layer structure for osteochondral repair, which could enhance the interlayer bonding through dynamic imine bonds and matrix continuity. Therefore, the multifunctional hydrogels have shown great promise and are expected to be an effective strategy for bone and cartilage regeneration.

### 4.2 Cardiac Tissue Repair

Myocardial infarction (MI) leads to irreversible cardiomyocyte loss and fibrotic remodeling, posing a significant burden in cardiovascular medicine [189]. Hydrogels have emerged as promising carriers for angiogenic factors, stem cells, and extracellular vesicles, creating a supportive microenvironment for cardiomyocyte survival and vascular regeneration. Conductive hydrogels, engineered with polypyrrole, PEDOT, or graphene derivatives, restore impaired electrical conductivity, synchronize cardiac contractions, and improve functional recovery after MI. Cheng et al. [190] developed an injectable pH‐responsive conductive hydrogel that could intelligently release the metformin and exosomes through the dynamic imine bonds, thereby activating pathways such as PI3K/AKT to enhance myocardial repair (Figure 10B). Injectable thermosensitive hydrogels further allowed the localized and minimally invasive delivery into infarct regions, while ROS‐scavenging and immunomodulatory hydrogel systems reduced the oxidative stress and modulated the macrophage polarization to limit adverse remodeling [191]. These multifunctional systems highlight the potential of multifunctional hydrogels as both structural and therapeutic scaffolds in cardiac regeneration.

### 4.3 Neural Tissue Regeneration

Neural tissues, including the central and peripheral nervous systems, have limited regenerative capacity following trauma or degenerative disease [192]. Hydrogels offer a versatile platform to promote neuroregeneration by providing guidance cues for axonal extension, support for Schwann cell proliferation, and delivery vehicles for neurotrophic factors such as NGF and BDNF. The aligned hydrogel scaffolds and gradient hydrogel scaffolds have shown particular promise in directing neurite outgrowth and restoring neural circuitry. For example, Cai et al. [193] constructed a GelMA‐MXene hydrogel nerve conduit with microgrooves through structural guidance, which enabled the directed growth and differentiation of neural stem cells, thereby enhancing the repair of spinal cord injuries. Furthermore, conductive hydrogels with carbon nanotubes or conductive polymers enabled the transmission of electrical signals, thereby supporting functional recovery in spinal cord injury models. Li et al. [194] synthesized acetylcholine/tyrosine‐modified chitosan derivatives, which were in situ polymerized with sodium alginate and pyrrole monomer to form conductive hydrogels. By adjusting the proportion of polypyrrole, they achieved a safe electrical conductivity of 1.82 × 10^−^^3^ S/cm to promote nerve repair (Figure 10C). In peripheral nerve repair, adhesive and self‐healing hydrogels could serve as nerve guidance conduits, bridging transected nerves and enhancing axonal regeneration. Therefore, with integration of cell therapy and bioelectronics, multifunctional hydrogel‐based neural scaffolds are advancing toward clinical translation for spinal cord and peripheral nerve injuries.

### 4.4 Organ Repair and Antifibrosis Applications

Beyond musculoskeletal and neural tissues, hydrogels are being engineered for hepatic, renal, and pulmonary tissue regeneration [195]. In chronic liver disease, antioxidant and antifibrotic hydrogels loaded with curcumin, resveratrol, or siRNA molecules can attenuate oxidative stress, inhibit stellate cell activation, and reduce collagen deposition. Liang et al. [196] designed a CCO/HGF@EV hydrogel, which eliminated the ROS and alleviated the oxidative damage in acute liver failure through the synergy of exosomes and hepatocyte growth factor (HGF), thereby promoting the regeneration outcomes (Figure 10D). For renal regeneration, bioactive hydrogels delivering growth factors such as VEGF can promote vascularization and functional nephron recovery [197]. In pulmonary fibrosis models, injectable hydrogels carrying anti‐inflammatory agents or gene‐editing tools provide localized therapy to minimize systemic toxicity. Evangelista‐Leite et al. [198] prepared a bioactive peptide hydrogel containing 224 proteins by decellularizing porcine lung extracellular matrix and digesting it with pepsin. This hydrogel not only regulated tissue repair and cell cytoskeleton pathways but also reduced the inflammatory, oxidative damage, and fibrosis markers in the bleomycin model, thereby providing a new mechanism for local treatment. So, the organ‐specific applications illustrate the adaptability of multifunctional hydrogels in addressing complex pathological microenvironments and emphasize their potential in systemic regenerative medicine.

### 4.5 Corneal Tissue Repair

Corneal disease can cause clouding, distortion, scarring, and eventually blindness, which is the second major cause of blindness. So, the development of polymeric hydrogel‐based scaffolds provides a suitable alternative for corneal repair and tissue regeneration. Most of the currently used biological materials differ in composition from the natural cornea, lacking the necessary biochemical components of the cornea. Over the past decade, research on corneal regenerative hydrogels mainly focused on simulating physiological characteristics and microenvironment regulation of corneal tissue to achieve a synergistic effect of optical function restoration and nerve regeneration [199]. In the design of biomimetic structures, studies have constructed three‐dimensional scaffolds with nanoscale pores through photopolymerization or electrospinning techniques. These scaffolds can simulate the unique collagen fiber arrangement of corneal stroma, thereby maintaining the transparency of the material and providing an ordered spatial guidance for epithelial cell migration [200, 201, 202]. The strategy of integrating bioactive factors is another core direction. By loading polypeptide molecules such as epidermal growth factor and basic fibroblast growth factor, or introducing seed cells such as limbal stem cells and mesenchymal stem cells, the hydrogels can activate signal pathways such as extracellular signal‐regulated kinase and phosphatidylinositol 3‐kinase, thereby promoting corneal epithelial cell proliferation and nerve axon regeneration [203, 204]. Additionally, to address the inflammatory microenvironment after the injury, the multifunctional hydrogels also need to integrate anti‐inflammatory, antioxidant, and antibacterial modules. For example, Barroso et al. [205] introduced vitamin C derivatives to eliminate reactive oxygen species or grafted the antibacterial peptides to inhibit the formation of pathogenic bacteria biofilms. These designs help reduce inflammation damage and lower the risk of infection. The collaborative application of these technical paths is gradually solving the problems of structural reconstruction and functional restoration after corneal injury, providing repair solutions for different types of corneal defects such as chemical burns and ulcerative keratitis.

### 4.6 Gastrointestinal Tissue Repair

The research focus of the gastrointestinal repair hydrogel lies in addressing the challenges of mucosal protection and targeted repair under dynamic physiological conditions [206, 207]. Its core technical path is centered on three major directions of environmental responsiveness, precise delivery, and immunoregulation. The environmental responsiveness design achieves intelligent adaptation by integrating pH‐sensitive polymers and temperature‐responsive materials. For example, sodium alginate could rapidly gel in the acidic environment of the stomach to form a physical barrier, while poly (N‐isopropylacrylamide) could undergo a phase transition under body temperature conditions. This dual‐response feature ensured that the hydrogel formed a stable protective layer in the stomach and released loaded active ingredients in the neutral intestinal environment [208, 209]. The targeted delivery system enhanced the repair efficiency through the receptor‐mediated and microenvironment‐responsive mechanisms. Li et al. reported a biodegradable hydrogel occlude with robust in vivo adhesion, which could seal the large gastric tissues via endoscopic delivery to the porcine stomach and monitor the healing process with improved retention of endogenous growth factors. In view of the simple hydrogel fabrication using molding technique, the biodegradable hydrogel occlude could be facilely tailored with various topologies according to application scenarios in surgical and minimally invasive endoscopic delivery, thus offering a promising alternative for clinical repair of gastrointestinal perforations and other organs [210]. In addition, the folic acid‐modified hydrogel particles could specifically bind to the high expression of folic acid receptors on the inflamed mucosa surface, while the cross‐linked network containing ROS‐sensitive bonds could break down at the inflammatory site due to the increase in reactive oxygen species concentration, achieving controlled and spatially controlled drug release [211, 212]. The immune regulation module is another key aspect of gastrointestinal repair. The hydrogel can polarize macrophages to the M2 phenotype by loading anti‐inflammatory factors such as IL‐10 and transforming growth factor β, or by carrying regulatory T cell exosomes, while promoting the expression of tight junction proteins. These effects collectively promote the reconstruction of the mucosal barrier and the restoration of stable immune microenvironments [213, 214]. Therefore, these technological innovations provide a new treatment paradigm for gastrointestinal mucosal injury, such as gastric ulcers and Crohn's disease, and promote breakthroughs in the application of hydrogel materials in dynamic physiological environments.

Taken together, these cross‐tissue applications demonstrate that multifunctional hydrogels are not confined to cutaneous wound healing but represent a broadly applicable platform for tissue engineering and regenerative medicine. By integrating bioactive delivery, mechanical reinforcement, immunomodulation, and stimuli‐responsive behavior, hydrogels can be customized to the physiological and pathological demands of distinct tissues. This versatility underscores their pivotal role in the future development of next‐generation regenerative therapies.

## 5 Conclusion and Prospects

The repair of injured tissues is a highly orchestrated and dynamic process influenced by multiple cellular, molecular, and environmental factors, and improper treatment methods have a significant impact on this recovery process. Multifunctional hydrogels, by virtue of their biomimetic architecture, tunable mechanics, and versatile delivery capabilities, have emerged as a cornerstone biomaterial system in tissue engineering and regenerative medicine. This review firstly provides a summary of the main effect factors influencing the cause of the wound, the depth of injury, and the duration of the healing process, as well as the therapeutic strategies and recent advancements of hydrogel wounds for skin regeneration. Then, as highlighted in this review, hydrogels have undergone a remarkable evolution from early‐generation wound dressings primarily to maintain a moist healing environment, to sophisticated multifunctional systems capable of immunomodulation, antimicrobial activity, hemostasis, controlled drug delivery, and dynamic responsiveness to the complex in vivo microenvironment. This transformation has enabled their application not only in cutaneous healing but also in bone and cartilage regeneration, cardiac repair, neural tissue engineering, organ anti‐fibrosis therapies, corneal repair, and gastrointestinal closure, thereby underscoring their adaptability across diverse physiological contexts.

Despite substantial progress, significant barriers remain before hydrogel‐based therapies can achieve widespread clinical adoption. First, a major consideration that warrants more detailed discussion is the degradation kinetics of hydrogels and their compatibility with clinical healing timelines, as unpredictable breakdown can result in toxic by‐products, incomplete resorption, or impaired integration with host tissue. While degradability is frequently cited as a necessary property, systematic data comparing degradation rates among different hydrogel classes remain limited. For example, natural polymer‐based hydrogels such as collagen, gelatin, chitosan, and hyaluronic acid typically degrade within days to weeks due to enzymatic cleavage, which may align with the early inflammatory and proliferative phases of skin wound healing but render them unsuitable for long‐term structural support in bone or cartilage repair [215, 216, 217, 218]. By contrast, synthetic hydrogels such as polyethylene glycol (PEG)‐based systems or polyvinyl alcohol (PVA) can exhibit degradation times extending from several weeks to months, offering durability but sometimes persisting longer than clinically desirable, potentially leading to foreign body reactions [219, 220, 221, 222, 223]. Hybrid or composite hydrogels have been designed to fine‐tune this window, yet standardized comparisons remain scarce. Clinically, the optimal degradation time should approximate the healing kinetics of the target tissue: approximately 2–3 weeks for superficial skin wounds, 6–8 weeks for deep dermal or muscle injuries, and several months for bone and cartilage regeneration [224, 225, 226, 227, 228, 229]. Hydrogels that degrade too quickly may collapse before adequate tissue integration occurs, whereas those that degrade too slowly may impede tissue remodeling or leave residual by‐products. Thus, the rational design of hydrogels with controllable, tissue‐specific degradation profiles is a central requirement for clinical translation. Future studies should prioritize generating quantitative degradation data under both in vitro and in vivo conditions, and directly correlate these results with functional outcomes in relevant large‐animal models. Second, the mechanical reinforcement is always insufficient, particularly in load‐bearing tissues such as cartilage and bone, where durable performance under physiological stress, such as the stomach and intestine, is essential. Third, biological safety and regulatory approval present hurdles, as complex hydrogel formulations incorporating nanoparticles, exosomes, or genetic materials raise questions regarding the immunogenicity, long‐term biocompatibility, and reproducibility. Fourth, the scalability of manufacturing remains unresolved, and current laboratory synthesis routes may be unsuitable for large‐scale production under GMP conditions, while cost‐effectiveness is essential for real‐world translation. Finally, reliance on rodent models still limits predictive accuracy, and large animal models with closer physiological similarity to humans are urgently needed, along with standardized evaluation frameworks for safety and efficacy.

Looking ahead, several promising avenues merit attention. From a materials science perspective, the design of “next‐generation hydrogels” with programmable degradation, bioactive reinforcement, and hierarchical architectures will allow tissue‐specific tailoring. From a biological standpoint, the combination of hydrogel scaffolds with stem cells, extracellular vesicles, or immunoregulatory molecules can synergistically enhance tissue regeneration and address the multifactorial nature of tissue injury. From a technological angle, the integration of hydrogels with bioelectronics, wearable devices, and 3D bioprinting technologies offers opportunities for real‐time monitoring, spatiotemporal control of therapeutic release, and patient‐specific scaffold design. From a clinical translation perspective, the establishment of standardized fabrication methods, reproducible characterization criteria, and cost‐effective manufacturing pipelines will be indispensable. At the same time, the regulatory guidelines should also be adjusted accordingly to accommodate the multifunctional and “living” biomaterials, while ethical considerations surrounding advanced therapies must be carefully addressed.

In conclusion, multifunctional hydrogel‐based platforms have demonstrated the potential to reshape the landscape of regenerative medicine by providing adaptive, intelligent, and customizable therapeutic solutions. Yet, realizing this potential will require not only scientific and engineering innovation but also multidisciplinary collaboration among material scientists, clinicians, bioengineers, and regulatory authorities. By bridging these gaps, bioactive hydrogels may ultimately transition from experimental prototypes to standardized clinical products, driving the next era of regenerative therapies that are both broadly applicable and tailored to patient‐specific needs.

## Author Contributions

Jieran Lyu, Xuemiao Liu, and Qiqi Yang were responsible for conceptualization, data curation, methodology, project administration, validation, and writing the original draft. Yuchang Zhang was responsible for the investigation and formal analysis. Xing Wang was responsible for validation, project administration, and supervision. All authors approved this manuscript for publication.

## Ethics Statement

The authors have nothing to report.

## Conflicts of Interest

The authors declare no conflicts of interest.

## Data Availability

The authors have nothing to report.
